# In-feed *Arthrospira (Spirulina) platensis* supplementation enhances breast yield in heat-stressed broilers through modulation of proteostasis network

**DOI:** 10.3389/fphys.2026.1912121

**Published:** 2026-09-09

**Authors:** Lulu Liu, Brooklee M. Roach, Ahmed E. Dhamad, Reagan Sims, Rohana Liyanage, Michael T. Kidd, Walter Bottje, Elisabeth S. Greene, Sami Dridi

**Affiliations:** 1 Center of Excellence for Poultry Science, University of Arkansas, Fayetteville, AR, United States; 2 Biology Department, College of Science, Wasit University, Wasit, Iraq; 3 Department of Agricultural Sciences, Texas State University, San Marcos, TX, United States; 4 Department of Chemistry and Biochemistry, University of Arkansas, Fayetteville, AR, United States

**Keywords:** breast yield, broilers, heat shock proteins, heat stress, protein synthesis, proteostasis, ubiquitin

## Abstract

Heat stress (HS) is a significant economic, welfare, and production burden to worldwide poultry production sustainability. Therefore, effective and sustainable mitigating strategies are needed. Driven by consumer’s changing preferences and demand for natural products, microalgae have attracted substantial attention from the poultry industry and have become the fastest growing segment of animal feed additives, yet their modes of action are still not well defined. This study aimed to determine the effect of *Arthrospira platensis* (*Spirulina*, SP) on growth performance in heat-stressed broilers and to delineate its underlying mechanisms by using *in vivo* and *in vitro* studies. Six hundred one-day old male Cobb500 chicks were randomly allocated to 12 environmental chambers (2 floor pens/chamber, 24 pens in total, 25 birds/pen) and fed two diets (standard control diet, C *vs. SP*-supplemented diet at 0.5% inclusion rate). On day 29, birds were exposed for two weeks to 2 environmental conditions (thermoneutral, TN, 24°C vs. cyclic HS 36°C, 8h/day). Growth performances, carcass parameters, and muscle myopathy incidences were determined. Chicken primary embryonic myoblasts (CPEM) were isolated, cultured, and treated with either benzoic acid (BA) or quercetin (QCT)-compounds abundant in *SP*- prior to a 6-h exposure to HS (45°C) or control conditions (37°C). Gene and protein expressions involved in protein proteostasis network (synthesis, degradation, and chaperonin) were measured by qPCR, immunoblot, and immunofluorescence. Data were analyzed by two-way ANOVA and Tukey test, and significance was set at *P* < 0.05. Heat stress significantly increased core body temperature (CBT) and water intake and depressed feed intake and growth performance in broilers. In-feed *SP* supplementation reduced CBT, improved water conversion ratio (WCR) by 36.5 points, and enhanced breast yield, without affecting muscle (woody breast and white striping) myopathies. Supplementation of *SP* significantly increased the phosphorylated levels of mTOR^Ser2481^, mTOR^Ser2448^, and S6K^Thr421/Ser424^, decreased p-eIF2α^Ser51^, ubiquitin, and HSP60/90 in heat-stressed broilers. Treatments of CPEM with BA or QCT recapitulated the effects of *SP*. Collectively, supplementation of *SP* enhanced breast yield in heat-stressed broilers through modulation of proteostasis network by enhancing protein synthesis (mTOR-S6K-eIF2α pathway), reducing protein degradation (ubiquitin machinery), and regulating chaperonins.

## Introduction

Despite advanced management technologies, cutting-edge equipment, and sophisticated poultry houses, heat stress (HS) still hinders poultry production (Lara and Rostagno, 2013; Brugaletta et al., 2022; Teyssier et al., 2022). The adverse effects of HS on poultry growth, reproduction, welfare, and meat quality are well documented (Goo et al., 2019; Abo Ghanima et al., 2020; Aloui et al., 2024) and its effect on bird’s physiology can vary from discomfort to organ(s) injury and in severe case to death (Huang et al., 2024). For instance, the Indian heat waves of summer (May) 2015 killed over 17 million chickens (Hadauria et al., 2016). In 2003, the European heat wave resulted in the death of more than one million chickens in France (Fouillet et al., 2008), which has been reproduced during the summer 2023 with a significant financial damage in the millions of euros. Heat stress caused heavy annual economic losses between $128 and $165 million to the U.S. poultry industry (St-Pierre et al., 2003). Taken together, HS is a significant worldwide economic, welfare, and production burden, and it will continue to threaten the poultry production sustainability as heat waves are predicted to become more frequent, intense, and widespread in the coming years (Tripathy et al., 2023; Murray et al., 2024; Shahvandi et al., 2024; Urban, 2024). There is, therefore, a critical need to identify effective and sustainable strategies to prevent and mitigate the deleterious effects of HS, while improving poultry growth and producer profitability.

Although they need to be improved and finetuned, seminal managerial, genetic, and nutritional research have produced significant strategies to alleviate HS in livestock and poultry (Azoulay et al., 2011; Van Goor et al., 2015; Cappelaere et al., 2021; Pecjak et al., 2022; Nawaz et al., 2023). Recently, fueled by consumers changing preferences, values, tastes, and demand for natural products, phytogenics and phycogenics have attracted substantial attention from the poultry and livestock industries, and have become the fastest growing segments of animal feed additives with a market value of approximately $1.34 billion in 2024 (Research, 2025). Due to its richness in amino acids, polyunsaturated fatty acids, essential minerals, vitamins, antioxidants, polyphenols, and pigments (Alfadhly et al., 2022; Bortolini et al., 2022; Mullenix et al., 2022; Alotaiby et al., 2024; Podgorska-Kryszczuk, 2024), *Arthrospira platensis (known as Spirulina, SP)*, a photosynthetic prokaryotic blue-green microalgae, gained recognition in various industries and globally consumed as a food supplement (Kay, 1991). It has been shown that *SP* supplementation enhances the antioxidant system and strengthens many immune processes in poultry (Qureshi and Ali, 1996; Qureshi et al., 1996; Wu et al., 2005). Furthermore, various studies have supported the ability of *SP* to improve growth performance, and feed efficiency in poultry (Yusuf et al., 2016; Pestana et al., 2020), yet the underlying molecular mechanisms are not well defined. It is worth mentioning that the majority of these studies, including our own (Velten et al., 2018; Mullenix et al., 2021, 2022; Spinola et al., 2024), focused on replacing soybean meals with *SP* in poultry diets, and the results were not always consistent. As the price of diet containing *SP* at high inclusion rate could be expensive, depending on origin, quality, production methods, certification, and packaging, we undertook the present study to determine: 1) the effect of *SP* as a feed additive at 0.5% inclusion rate on broilers growth performance, and 2) delineate its mode of action.

## Methods

### Bird experiments

One-day old male Cobb500 chicks (n = 600) were obtained from Cobb-Vantress hatchery (Siloam Springs, Arkansas) and randomly allocated to 12 environmental chambers in the Poultry Environmental Research Laboratory at the University of Arkansas (2 floor pens/chamber, 24 pens in total, 25 birds/pen). Each pen was covered with clean pine wood shavings and equipped with separate commercial hanging feeders and individual drinkers. Temperature was maintained at 32 °C for the first 3 days, and then gradually reduced by 3 °C each week until it reached 24 °C on day 21. The lighting program was 24 h light for the first 3 days, reduced to 23 h light:1 h dark from day 4 to 7, and then reduced further to 18 h light:6 h dark thereafter. Birds were fed either a standard diet (C), formulated to meet or exceed broiler requirements (**Supplementary Table 1**), or the standard diet supplemented with 0.5% *SP* (Spirulina Powder-organic, Cat # RFI-GO130001M, FRI Ingredients, Broomfield, CO). The dose of SP was chosen based on pilot and previous studies (Mullenix et al., 2021, 2022). On Day 29, birds were exposed to two environmental conditions: thermoneutral (TN, 24 °C) or chronic cyclic heat stress (HS, 36 °C for 8 h/day from 8:30 am to 4:30 pm) to mimic summer season in Arkansas, USA. Day 29 marks the start of the finisher phase, initiating the HS treatment period to account for increased metabolic heat production. To evaluate the effects of feed depression associated with HS, a pair fed (PF) group was included, which received an equivalent amount of feed as the HS group consumed but remained in TN conditions. The diet and environmental factors resulted in a total of 6 treatments in a 2 × 3 factorial design (4 pens/treatment, 100 birds/treatment). One day before the onset of HS, two chickens per pen were randomly selected and a Thermochron temperature logger (iButton, Embedded Data Systems, KY) was placed in the crop via oral gavage for continuous monitoring of core body temperature as previously described (Greene et al., 2021b). Body weights were recorded weekly, and feed and water intake were measured on a daily basis from day 1 to day 42 and corrected for mortality. On day 41, the 2 birds per pen containing the Thermochron temperature loggers were euthanized via cervical dislocation, and breast tissues (*Pectoralis major*) were aseptically collected from the same cranial area, snap-frozen in liquid nitrogen, and stored at -80°C until further analysis.

### Processing

On day 44 of age and after 12h of feed withdrawal, 10 birds/pen were randomly selected, and wing tagged. Birds were weighed and processed using a commercial inline system at the University of Arkansas Pilot Processing Plant (Fayetteville, AR, United States) as previously described (Orlowski et al., 2018; Maynard et al., 2023). Birds were electrically stunned, exsanguinated, scalded, and defeathered using commercial inline equipment. After mechanical evisceration, abdominal fat was manually removed from each bird and both fat pad and hot carcass were weighed. Carcasses were then chilled via immersion in chilling tanks and held at 0 °C for 3 h and then were weighed again and deboned to determine the weight and the yield (relative to live weight) of breast (*Pectoralis major*), tender (*Pectoralis minor*), wing, and leg quarter.

### Muscle myopathies scoring and categories

The incidences of muscle myopathies, woody breast (WB) and white striping (WS), were single-blind assessed and macroscopically scored by a well-trained person as previously described (Greene et al., 2019; Cauble et al., 2020; Greene et al., 2025a). Based on breast hardness and texture, WB was classified into various 3 point-based grades and categories [normal (NORM, score 0); moderate (MOD, score 1) and severe (SEV, score 2-3)]. White striping, however, was sorted based on the white striation coverage into three groups (NORM, score 0; MOD, score 1; and SEV, score 2-3).

### Isolation and *in vitro* culture of chicken primary embryonic myoblasts (CPEM)

The isolation and culture of CPEM were performed as described earlier by our group (Greene et al., 2023, 2025). At 80-90% confluence, cells were pre-treated with quercetin (QCT, 10μM) or benzoic acid (BA, 50μM) for 1h and untreated cells were used as controls. Cells were further kept at 37°C or exposed to HS (45°C) for 6h, which resulted in a 2x2 factorial design experiment with environment and treatment as independent variables. The doses of QCT and BA were selected based on pilot and previous studies (Yilmaz et al., 2009; Flees et al., 2017; Wang et al., 2017). The reagents QCT (Cat. N0. Q4951) and BA (Cat. N0. 242381) were purchased from Sigma (St. Louis, MO).

### RNA isolation, reverse transcription, and real-time quantitative PCR

Total RNA extraction and RT-qPCR were performed as previously described (Dridi et al., 2012; Vignale et al., 2015; Greene et al., 2023). Briefly, Total RNA was extracted from breast muscle samples and CPEM cells using TRIzol Reagent (Thermo Fisher Scientific, Waltham, MA) according to the manufacturer’s instructions. cDNA was synthesized from 1 μg of total RNA using qScript cDNA Synthesis kit (Quantabio, Beverly, MA). qPCR was performed using a 2× Universal SYBR Green fast qPCR Mix (ABclonal, Woburn, MA) on 7500 Real Time PCR system (Applied Biosystems, Foster city, CA) with chicken specific primers for target genes summarized in **Table 1**. Primer efficiencies (91–101%) were validated using a 6-point serial dilution and standard curves with SYBR Green Master Mix. The relative expression of target genes was calculated using 2^-ΔΔCt^ method (Schmittgen and Livak, 2008) and ribosomal 18S as a housekeeping gene. Control diet-TN condition or untreated cells-37°C were used as calibrators.

**Table 1:** Oligonucleotide QPCR primers.

Gene^1^	Accession number^2^	Primer sequence (5’ → 3’)	Orientation	Product size (bp)
*mTOR*	XM_417614	CATGTCAGGCACTGTGTCTATTCTC	Forward	77
		CTTTCGCCCTTGTTTCTTCACT	Reverse	
*RPS6KA1*	NM_001109771	GTCAGACATCACTTGGGTAGAGAAAG	Forward	60
		ACGCCCTCGCCCTTGT	Reverse	
*RPS6KA2*	XM_040666539	CTGCGAGCCCTCTTCAAAAG	Forward	65
		TCTTCCACTCCATCGAATCCA	Reverse	
*RPS6KA3*	XM_040661003	ACTCCAAAAGATTCGCCTGGTA	Forward	60
		TCCGCGAAAAAGCTGATGT	Reverse	
*RPS6KA5*	NM_001012587	CAAAGACACTGCAGCCAACAA	Forward	113
		TTATAACCACGCAAATCTTCAAGACA	Reverse	
*RPS6KA6*	XM_040670295	CAGAACTGGCCCTTGCTTTG	Forward	67
		TCTGGCTTCAGGTCCCTGTATAC	Reverse	
*RPS6KB1*	NM_001030721	CCTTGGCATGGAACATTGTG	Forward	72
		CGGATCTTTTCTGGACCTCTGT	Reverse	
*RPS6KB2*	XM_046919053	CCGTGACCTCAAGCCAGAAA	Forward	66
		CCGAAGTCCGTCAGCTTGAT	Reverse	
*RPS6KC1*	XM_004935322	CCATCCATTTTTTGCCCTCATA	Forward	59
		GCGAGCGCATCTCTCATTTC	Reverse	
*Eif2α*	NM_205368	TCCTGGTGAAGACTGGAACCA	Forward	59
		CATGACCCGCATGTTGTGA	Reverse	
*EiF4EBP1*	XM_424384	CTCTCCGTGTGGGTGTGAATAC	Forward	56
		CCCCACAGCCCATCATCA	Reverse	
*PSMA1*	NM_205020	GCTGCCGGCACAGGAA	Forward	55
		ACACCGTCACGTCGTTATCG	Reverse	
		CTTCAACGGCGGGACTGT	Forward	
*PSMB1*	NM_001007905	CAGAGGCAACAATGCAGAAGTCT	Reverse	59
		GGTTCGGAGCTCATCCAGAA	Forward	
*PSMC1*	NM-204958	AGTTCGCGCACCAGCTTT	Reverse	56
		CGCAGGCTGGACATCTTTG	Forward	
*PSMD1*	NM_001012600	CAGCATCCCAGGAACATCGT	Reverse	60
		GCTCCAACTGCAAAGCCTCTA	Forward	
*PSMF1*	NM_00103158	GGTAACGCCAGGAATTCATTG	Reverse	63
		AGGCTGCACAATCCGTAACAC	Forward	
*UBA2*	NM-001030571	AACAAATACTTGGCCCACACAA	Reverse	66
		AGCTCAAGCAGAGGCGTACAC	Forward	
*UBE2*	NM_001396822	CGAACCCTCTTTTCGTATTCCA	Reverse	63
		CCACGCGCCCTTAAAGC	Forward	
*POMP*	XM_040658373	GACGCTGGGCCTGTTTCA	Reverse	56
		TGCAACAATTTAACCAACGATACCT	Forward	
*WWP1*	NM_001012554	GGCCCAAGTGGATCGTTCT	Reverse	70
		GGGAGTCCCAGCCTGTGA	Forward	
*CLTC*	NM_001080117	GCTTTGCATCAGTACGGTAGTTGA	Reverse	84
		AGTGATGACGAAGCTGGGAGAT	Forward	
*AMPH*	NM_001004398	GGTGCCCCTTGAATTGTGAA	Reverse	60
		GCCAGCTTTCTGCTCTTGTACA	Forward	
*SH3GL2*	NM_204530	AGGATCTGTGTGGCCTGCTT	Reverse	61
		ACCTCGTCAATATGGGCATCA	Forward	
*FLOT1*	XM_040694369	TCGTCGTGGATGTCCTTCAG	Reverse	58
		TGCAAGAAGGAAATGTTGGATGT	Forward	
*FLOT2*	NM_001030719	GCACGCCTGGAATCTGCTAT	Reverse	65
		ACACATTCTGCGACCCTCTGT	Forward	
*CAV1*	NM_001105664	GGCTCGGATGCTGCTGAA	Reverse	59
		GAGCTGCAGAATGCCCTTACTC	Forward	
*CAV2*	NM_001007086	CTTAATGTGCAAAAAATACGCAGTGT	Reverse	70
		TCGAGGCGCTCTCCAAGA	Forward	
*CAV3*	NM_204370	AGGTTTCCTTCCGCAGCATA	Reverse	57
		TCCCCTCCCGTTACTTGGAT	Forward	
*r18S*	AF173612	GCGCTCGTCGGCATGTA	Reverse	60

^1^ AMPH, amphiphysin;CAV, caveolin; CLTC, Clathrin heavy chain; EiF2α, eukaryotic translation initiation factor 2 alpha; EiF4EBP1, eukaryotic translation initiation factor 4E binding protein 1; FLOT, flotillin; mTOR, mechanistic target of rapamycin; POMP, proteasome maturation protein; PSMA1, proteasome 20S subunit alpha 1; PSMB1, proteasome 20S subunit beta 1; PSMC1, proteasome 26S subunit, ATPase 1; PSMD1, proteasome 26S subunit, non-ATPase 1; PSMF1, proteasome inhibitor subunit 1; r18S, ribosomal 18S; RPS6KA, ribosomal protein S6 kinase alpha; RPS6KB, ribosomal protein S6 kinase beta; RPS6KC1, ribosomal protein S6 kinase delta-1; SH3GL2, SH3 domain containing GRB2 like 2; UBA2, ubiquitin like modifier activating enzyme 2; UBE2, ubiquitin conjugating enzyme E2; WWP1, WW domain containing E3 ubiquitin protein ligase 1.

^2^ Accession number refer to Genbank (NCBI).

### Western blot analysis

Proteins were isolated from muscle tissues and CPEM cells, quantified, and subjected to immunoblot analysis as previously described (Dridi et al., 2012; Vignale et al., 2015). Primary antibodies used were: rabbit anti-mechanistic target of rapamycin (mTOR, #2972, Cell Signaling Technology, Danvers, MA), rabbit anti-phospho-mTOR^Ser2481^ (#AP0978, ABclonal, Woburn, MA), rabbit anti-phospho-mTOR^Ser2448^ (#AP0115, ABclonal, Woburn, MA), rabbit anti-p70 S6 kinase α (S6Kα, #sc-230, Santa Cruz Biotechnology, Dallas, TX), rabbit anti-phospho-p70 S6 kinase α^Thr 421/Ser 424^ (p-S6Kα, #sc-7984-R, Santa Cruz Biotechnology, Dallas, TX), rabbit anti-phospho S6 ribosomal protein^Ser235/236^ (p-S6, #AP0538, ABclonal, Woburn, MA), rabbit anti-phospho eukaryotic translation initiation factor 2 alpha^Ser51^ (p-eIF2α, #AP0692, ABclonal, Woburn, MA), rabbit anti-eIF2α (#A0764, ABclonal, Woburn, MA), rabbit anti-ubiquitin (#A19686, ABclonal, Woburn, MA), mouse anti-heat shock protein 70 (HSP70, #MA3-007, Thermo Scientific, Waltham, MA), mouse anti-HSP90 (#ab59459, Abcam, Waltham, MA), and rabbit anti-HSP60 (#ARP61107_P050, Aviva Systems Biology San Diego, CA). Protein loading and normalization were assessed by Ponceau S staining and/or immunoblotting with the use of rabbit anti-vinculin (#V4139, Sigma, St. Louis, MO). Pre-stained molecular weight marker (Precision Plus Protein Dual Color) was used as a standard (Bio-Rad Laboratories, Hercules, CA). Protein bands were visualized using SuperSignal West Femto Maximum Sensitivity Substrate (Thermo Scientific, Waltham, MA) and captured by FluorChem M MultiFluor System (ProteinSimple, Santa Clara, CA). Image acquisition and analysis were performed with the use of AlphaView software (Version 3.4.0, ProteinSimple, San Jose, CA).

### Immunofluorescence staining

Immunofluorescence staining was performed as previously described (Dridi et al., 2012; Dhamad et al., 2020). Briefly, cells were grown in chamber slides and fixed with methanol for 20 min at −20 °C. Cells were blocked with serum-free protein block (Dako, Carpinteria, CA) for 1 h at room temperature, and then incubated with rabbit anti-phospho-mTOR^Ser2481^ (#AP0978, ABclonal, Woburn, MA) or rabbit anti-phospho-p70 S6 kinase α^Thr 421/Ser 424^ (#sc-7984-R, Santa Cruz Biotechnology, Dallas, TX) primary antibodies (1:200, in Antibody Diluent, Dako, Carpinteria, CA) overnight at 4 °C. The signal was visualized with DyLight 488-conjugated secondary antibody (Thermo Fisher Scientific, Grand Island, NY). Slides were cover-slipped with a Vectashield with DAPI (Vector Laboratories, Burlingame, CA), and images were obtained and analyzed using Zeiss Axio Imager M2, the Hamamatsu C11440 digital camera, and Zen Blue v2.5 software (Carl Zeiss Microscopy).

### Diet total phenolic content

Total phenolic content in the diet was determined as previously described (Greene et al., 2021a). Briefly, approximately 25 mg of control (C) and algae-containing (*SP*) diets were sonicated and mixed with 1 mL of methanol/water/formic acid (60:37:3 v/v/v) for 2 h, followed by centrifugation (9,223g; 10 min). The extraction was repeated with 1 mL of acetone/water/acetic acid (70:29.5:0.5 v/v/v) for 30 min followed by a second centrifugation (9,223g; 10 min). The extracts were dried in a nitrogen evaporator (Organomation, Berlin, MA) and were reconstituted with 0.5 mL of methanol/water/formic acid (60:37:3 v/v/v). The total phenol amounts in the extracts were determined using a modified Folin–Ciocalteu assay (Slinkard and Singleton, 1977). Gallic acid (GA) was used as a standard reference, and the results were expressed as mg of GA equivalent (GAE) per g of sample.

### Diet DPPH radical scavenging activity

The free radical scavenging activity of extracts was measured using the stable radical 2,2-diphenyl-1-picrylhydrazyl (DPPH) colorimetric assay as previously described (Greene et al., 2021a). Briefly, the extracts from C and *SP* diets were diluted (15:1) using methanol and centrifuged at 6,037g for 5 min. Supernatants (10 μL) was mixed with DPPH (140 μL, 0.1 mM), obscurantely agitated for 30 min, and the absorbance was measured at 517 nm using Synergy HT multimode microplate reader (BioTek, Shoreline, WA) following an adapted protocol from Akkari and colleagues (Akkari et al., 2016). The antioxidant activity was determined by using the scavenging effect of the Trolox standard curve and expressed as micromoles of Trolox equivalent (TE) per g of sample.

### LC-MS/MS analysis of flavonoids and phenolic acids in *SP*

The *SP* extracts were obtained using two different solvents, water (aqueous) and methanol as previously described (Riyadi et al., 2023; Salazar-Gonzalez et al., 2024). After filtration, centrifugation (12,000g, 10 min, 4°C), and passages through Strata C18-U column (8B-S002-JCH, Phenomenex, Torrance, CA), the extract eluted in 1 mL water or methanol and sent for LC-MS/MS analysis.

LC–MS/MS analysis was performed using a Shimadzu UPLC-20A/LC-30A system coupled to a Shimadzu 8060 triple quadrupole mass spectrometer equipped with a heated electrospray ionization (HESI) source operating in both positive and negative ion modes. Chromatographic separation was carried out using an ACQUITY Premier HSS T3 column with VanGuard FIT (1.8 µm, 2.1 × 150 mm), in combination with a C18 guard column (2.1 × 50 mm, 1.9 µm particle size). The mobile phase consisted of 0.1% formic acid (FA) in HPLC-grade water (solvent A) and 0.1% FA in methanol (solvent B). Flavonoids were separated using a linear gradient starting at 60% methanol and increasing to 100% methanol over 10 minutes. Phenolic compounds were separated using a linear gradient starting at 40% methanol and increased to 100% methanol over 10 minutes. The flow rate was maintained at 0.25 mL/min, and the injection volume was 1 µL. Multiple reaction monitoring (MRM) transitions for each standard were fully optimized using Shimadzu LabSolutions software (Version 5.89). For each analyte, the three most intense MRM fragment ions were selected. The most intense fragment ion was used as the quantifier (quant) ion, while the other two fragment ions were used as qualifier ions for confirmation, together with retention time matching.

### Statistical analysis

Data were analyzed by a two-way ANOVA with fixed factors of Diet (C *vs. SP* for chickens and C *vs.* BA or QCT treatments for CPEM) and environment (TN *vs.* HS *vs.* PF for chickens and 37°C *vs.* 45°C for CPEM). When the interaction was significant, means were compared by the *post hoc* multiple comparison Tukey HSD test. If the Diet or treatment × Environment interaction was not significant, the significant main effects were analyzed with separate Student t tests or one-way ANOVA as appropriate. The alpha level was 0.05. All analyses were run in GraphPad Prism version 10.4.2 (GraphPad Software, San Diego, CA), and results are reported as mean ± SEM.

## Results

### Antioxidant activity, total phenolic-, flavonoids- and phenolic acids-content in diet and SP

The DPPH assay showed that the *SP* diet exhibited a significantly higher antioxidant capacity compared to the control diet (11.47 ± 0.5 μM TE/g *vs.* 10.10 ± 0.1 μM TE/g, *P* = 0.0362, η^2^ = 0.5461, CI = 0.1223 to 2.618), however the total phenolic content did not differ between the two diets as determined by the Folin-Ciocalteau test (2.02 ± 0.03 mg CAE/g vs. 2.01 ± 0.09 mg CAE/g, *P* = 0.8481, η^2^ = 0.0066, CI= -0.1123 to 0.1323). Analysis of individual flavonoids and phenolic acids by LC–MS/MS showed that *SP* contains flavonols (quercetin, isorhamnetin, (+)-Catechin Hydrate) and derivatives, flavanones (pinotrobin, sakuranetin, hesperetin), and flavones (luteolin) (**Supplementary Table 2**). The phenolic compounds are mainly benzoic acids and derivatives (isovanillic acid, vanillic acid, gallic acid, hippuric acid, protocatechuic acid, salicylic acid), hydroxycinnamic acid (sinapic acid, chlorogenic acid, neochlorogenic acid(-)), and phenylacetic acid (homogentisic, m-hydroxyphenylacetic acid) (**Supplementary Table 2**).

### *In feed-SP* modulates core body temperature of heat-stressed broilers

**Figure 1:**
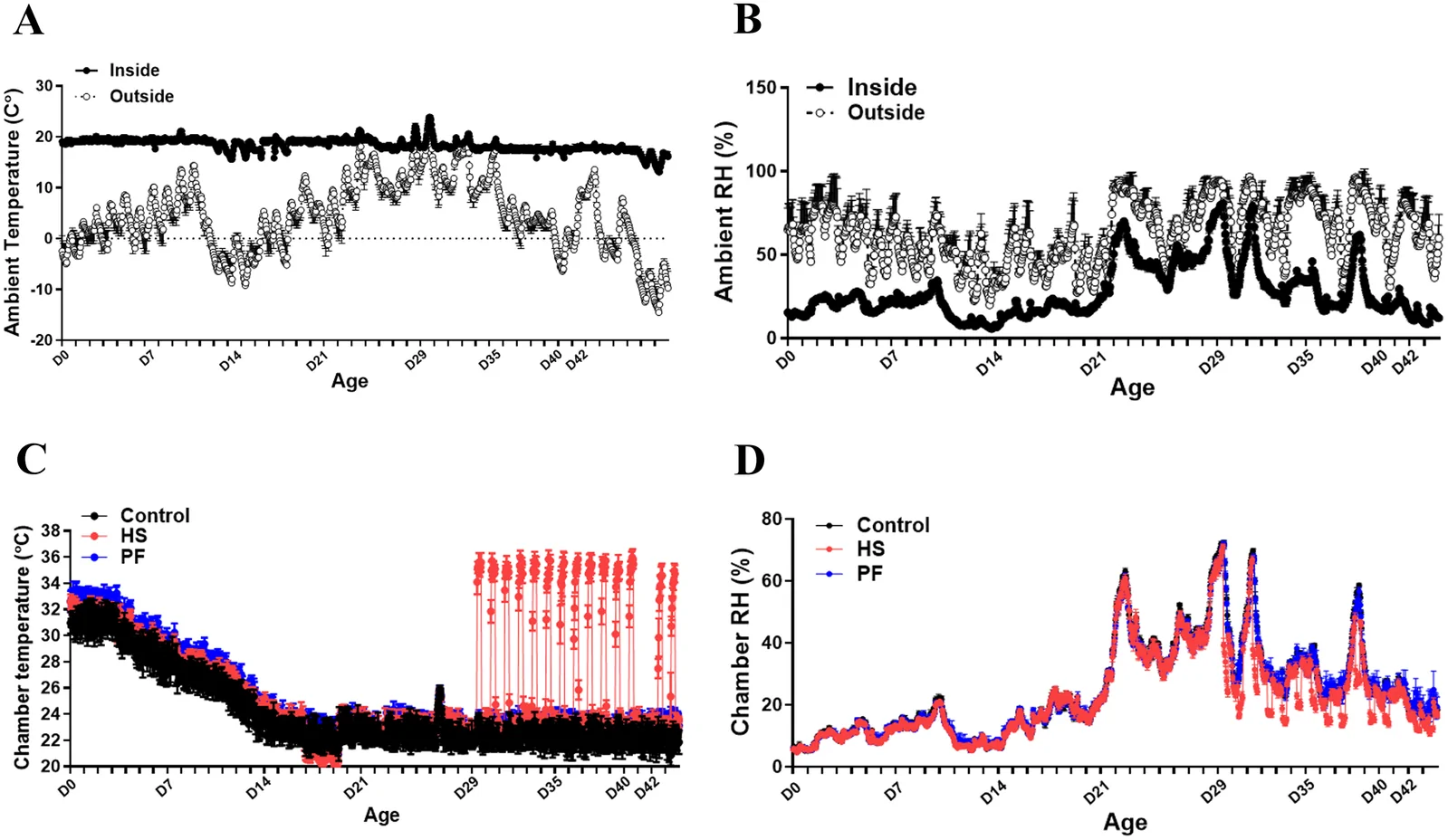
Environmental conditions during the experimental trial. Temperatures **(A, C)** and relative humidity [RH, **(B, D)**] fluctuations outside and inside of the environmental chambers during the cyclic heat stress experiment. Cobb500 chicks were raised under recommended conditions from day 0-28. From day 29-43, birds either raised at thermoneutral temperature (24°C) or exposed to chronic cyclic heat stress (36 °C for 8h/day from 8:30 am to 4:30 pm), except on day 41 birds were maintained at TN due to snowstorm. Data are presented as mean ± SD (n = 6 chambers/environmental condition).

The experiment was carried out from January 8^th^ to February 20^th^, 2025, for 44 days. The weathercast (temperature and RH) was monitored daily and is shown in **Figures 1A, B**. Typical to the Arkansas mild and four-season weather, the average outside temperature and RH varied approximately from -17.5°C to 24.6°C and 25% to 98.5%, respectively. The average temperature inside the barn (outside of the environmental chambers) was maintained at roughly 20°C, however the RH diverged from 7% to 79.9% (**Figures 1A, B**). As planned, the temperature inside of the environmental chambers was accurately controlled to achieve 36°C (HS) from 8:30 am to 4:30 pm (8h/day) and returned to 24°C (TN) during the rest of the time each day from day 29 to 42, resulting in cyclic HS conditions (**Figure 1C**). The RH inside the environmental chambers extended from 3.7% to 65.6% (**Figure 1D**).

**Figure 2:**
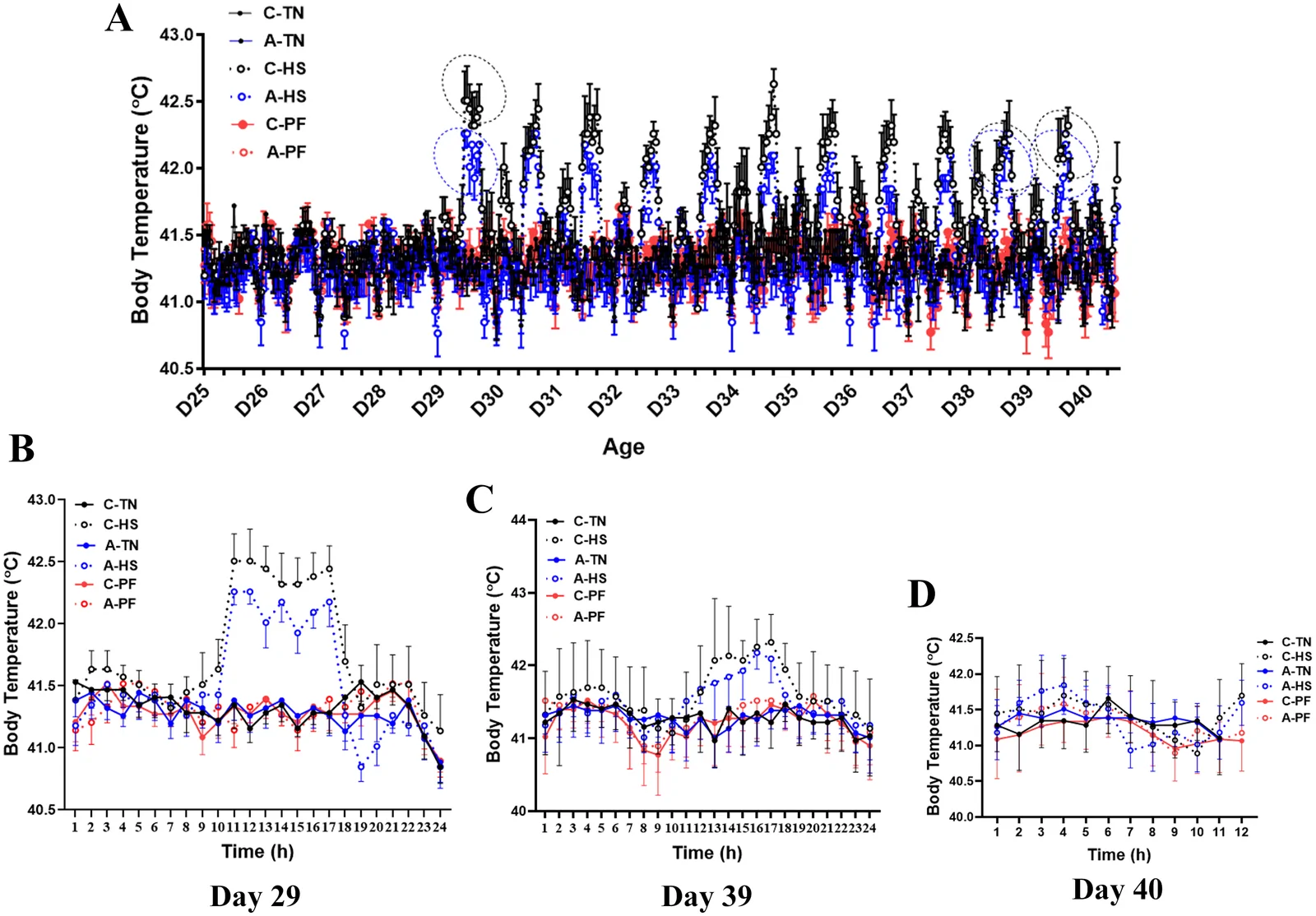
Core body temperature fluctuations during chronic cyclic heat stress (HS) experiment. **(A)** represents the period from d25-d40, including that of HS (d29-40). **(B-D)** represents a detailed daily variation from day 29, 39, and 40, respectively. Birds were exposed to two environmental conditions: TN, 24°C or HS (36°C, 8h/d) in a 2x2 factorial design as described in materials and methods. C-HS, control heat stress; C-PF, control pair fed; C-TN, control thermoneutral; A-TN, algae thermoneutral; A-HS, algae heat stress; A-PF, algae pair fed.

As anticipated, HS significantly increased CBT by ∼1.357 °C on day 29 compared to TN conditions, and this amplitude was gradually reduced to reach 0.5°C by day 40 (**Figures 2A–D**). The in-feed supplementation of *SP* reduced the CBT of heat-stressed broilers by 0.25°C on day 29 compared to control diet, and this effect was diminished as the experiment progressed (**Figures 2A–D**). Pair-feeding did not affect the CBT and birds maintained steady temperature (**Figures 2A–D**).

### *In-feed SP* improves growth performance of heat-stressed broilers

There was no significant environment by diet interaction for body weight (BW), average daily gain (ADG), cumulative feed intake (CFI), cumulative water consumption (CWC), feed conversion ratio (FCR) and water conversion ratio (WCR) (**Table 2**). As expected, HS significantly reduced CFI, BW, and ADG but it increased CWC, FCR and WCR (**Tables 2**, **3**). In-feed *SP* supplementation improved WCR by 36.5 (*P* = 0.0297, η^2^ = 0.0065, CI= -0.6939 to -0.0361) and 9.7 points (*P* = 0.0990, η^2^ = 0.0037, CI= -0.2123 to 0.0183) during HS (d29-42) and the whole experimental period (d1-42), respectively (**Table 3**). For the carcass, there was no significant diet by environment interaction for all measured parameters, except the tender yield (**Table 4**). Chronic HS exposure significantly decreased hot carcass, CWOG, and breast, but increased that of fat pad content (**Table 4**). Supplementing diets with *SP* increased breast yield (*P* = 0.0494, η^2^ = 0.0060, CI= -0.0140 to 1.014) and hot carcass yield (*P* = 0.0860, η^2^ = 0.0049, CI= -0.0695 to 1.070)) (**Table 4**). Heat stress reduced the severity of WB myopathy and administration of *SP* did not affect muscle myopathy (WB and WS) incidence (**Table 5**).

**Table 2:** Effect of *S. Plantensis* on growth performance of heat-stressed broilers.

Environment (E)^1^	TN		HS		PF		P-value		
Growth^2^/Diet (D)^3^	C	SP	C	SP	C	SP	E	D	E x D
BW (g/bird)									
D1-28	2,027 ± 29	1,997 ± 14	2,013 ± 24	1,974 ± 16	2,056 ± 21	2,048 ± 27	0.0512	0.1837	0.7827
D29-42	1,843 ± 39	1,877 ± 36	1,593 ± 24	1,717 ± 43	1,672 ± 49	1,708 ± 18	**0.0001**	**0.0443**	0.391
D1-42	3,870 ± 55	3,874 ± 45	3,606 ± 37	3,690 ± 45	3,728 ± 29	3,756 ± 37	**0.0002**	0.2725	0.6292
ADG (g/bird/d)									
D1-28	72 ± 1	71 ± 1	72 ± 1	70 ± 1	73 ± 1	73 ± 1	0.0512	0.1837	0.7827
D29-42	123 ± 3	125 ± 2	106 ± 2	114 ± 3	111 ± 3	114 ± 1	**0.0001**	**0.0445**	0.3984
D1-42	92 ± 1	92 ± 1	86 ± 1	88 ± 1	89 ± 1	89 ± 1	**0.0002**	0.2725	0.6292
CFI (g/bird)									
D1-28	2,587 ± 43	2,577 ± 22	2,591 ± 37	2,551 ± 24	2,626 ± 34	2,623 ± 33	0.2566	0.5186	0.832
D29-42	3,156 ± 40	3,159 ± 26	2,995 ± 30	3,036 ± 14	3,099 ± 36	3,059 ± 17	**0.0005**	0.958	0.3871
D1-42	5,949 ± 60	5,935 ± 40	5,788 ± 63	5,783 ± 28	5,931 ± 63	5,884 ± 50	**0.0191**	0.6121	0.9173
CWC (g/bird)									
D1-28	5,523 ± 44	5,440 ± 105	5,481 ± 80	5,469 ± 38	5,506 ± 76	5,714 ± 125	0.221	0.5904	0.2228
D29-42	7,523 ± 107	7,146 ± 190	9,410 ± 354	9,166 ± 196	7,501 ± 79	7,255 ± 132	**0.0001**	0.091	0.9298
D1-42	13,434 ± 101	12,949 ± 245	15,280 ± 426	15,002 ± 209	13,403 ± 135	13,340 ± 53	**0.0001**	0.1587	0.6623
FCR									
D1-28	1.277 ± 0.004	1.297 ± 0.004	1.292 ± 0.005	1.297 ± 0.007	1.286 ± 0.009	1.287 ± 0.010	0.5199	0.1402	0.3631
D29-42	1.555 ± 0.024	1.526 ± 0.018	1.687 ± 0.015	1.583 ± 0.046	1.661 ± 0.048	1.616 ± 0.015	**0.0082**	**0.0324**	0.4627
D1-42	1.393 ± 0.006	1.389 ± 0.007	1.439 ± 0.010	1.423 ± 0.013	1.438 ± 0.012	1.412 ± 0.006	**0.0011**	0.0658	0.5382
WCR									
D1-28	2.919 ± 0.024	2.921 ± 0.041	2.926 ± 0.021	2.962 ± 0.026	2.890 ± 0.055	2.988 ± 0.071	0.846	0.2166	0.5479
D29-42	4.085 ± 0.060	3.813 ± 0.135	5.904 ± 0.190	5.332 ± 0.046	4.502 ± 0.170	4.250 ± 0.096	**0.0001**	**0.0026**	0.3951
D1-42	3.356 ± 0.030	3.235 ± 0.058	4.030 ± 0.060	3.916 ± 0.037	3.472 ± 0.039	3.415 ± 0.027	**0.0001**	**0.0136**	0.731

^1^ HS, heat stress; PF, pair fed; TN, thermoneutral. ^2^BW, body weight; BWG, body weight gain; CFI, cumulative feed intake; CWC, cumulative water consumption; FCR, feed conversion ratio; WCR, water conversion ratio. ^3^C, control diet; SP, *spirulina Plantensis.*Bold values indicate significant P values.

**Table 3:** Main Effect of diet or environment on growth performance of heat-stressed broilers.

Main effect of environment					
Growth^1^/Environment^2^	TN	HS		PF	P-value
BWG (g/bird)					
D29-42	1860 ± 37.5^a^	1655 ± 33.5^b^		1690 ± 33.5^b^	**< 0.0001**
D1-42	3872 ± 50.0^a^	3648 ± 41.0^b^		3742 ± 33.0^ab^	**0.0008**
ADG (g/bird/d)					
D29-42	124 ± 2.5^a^	110 ± 2.5^b^		112.5 ± 2^b^	**<0.0001**
D1-42	92 ± 1.0^a^	87 ± 1.0^b^		89 ± 1.0^ab^	**0.0019**
CFI (g/bird)					
D29-42	3157.5 ± 33.0^a^	3015.5 ± 22.0^b^		3079 ± 26.5^ab^	**0.0013**
D1-42	5942 ± 50.0	5785.5 ± 45.5		5907.5 ± 56.5	**0.074**
CWC (g/bird)					
D29-42	7334.5 ± 148.5^a^	9288 ± 275^b^		7378 ± 105.5^a^	**<0.0001**
D1-42	13191.5 ± 173^a^	15141 ± 317.5^b^		13371.5 ± 94^a^	**<0.0001**
FCR					
D29-42	1.540 ± 0.021^a^	1.635 ± 0.030^b^		1.6385 ± 0.03^b^	**0.0202**
D1-42	1.391 ± 0.006^a^	1.431 ± 0.011^b^		1.425 ± 0.009^b^	**0.0044**
WCR					
D29-42	3.949 ± 0.097^a^	5.618 ± 0.118^b^		4.376 ± 0.133^c^	**<0.0001**
D1-42	3.295 ± 0.044^a^	3.973 ± 0.048^b^		3.443 ± 0.033^c^	**<0.0001**
Main effect of diet					
Growth^1^/Diet^3^	C		SP		P-value
BWG (g/bird)					
D29-42	1702.66 ± 37.33		1767.00 ± 32.33		0.193
D1-42	3734.66 ± 40.33		3773.33 ± 42.33		0.9819
ADG (g/bird/d)					
D29-42	113.33 ± 2.66		117.66 ± 2.00		0.1936
D1-42	89.00 ± 1.00		89.66 ± 1.00		0.6409
CFI (g/bird)					
D29-42	3083.33 ± 35.33		3084.66 ± 26.33		0.9759
D1-42	5889.33 ± 62.00		5867.33 ± 42.66		0.7701
CWC (g/bird)					
D29-42	8144.66 ± 180.0		7855.66 ± 172.66		0.247
D1-42	14039.0 ± 220.66		13763.66 ± 169.00		0.3222
FCR					
D29-42	1.634 ± 0.029		1.575 ± 0.026		0.1303
D1-42	1.423 ± 0.009		1.408 ± 0.008		0.2133
WCR					
D29-42	4.830 ± 0.14		4.465 ± 0.092*		**0.0297**
D1-42	3.619 ± 0.043		3.522 ± 0.040		0.099

^2^ ADG, average daily gain; BWG, body weight gain; CFI, cumulative feed intake; CWC, cumulative water consumption; FCR, feed conversion ratio; WCR, water conversion ratio. ^2^HS, heat stress; PF, pair fed; TN, thermoneutral. ^3^C, control diet; SP, *Spirulina Plantensis*.Bold values indicate significant P values.

**Table 4:** Effect of *SP* on carcass parameters yield (%) of heat-stressed broilers.

Environment (E)^1^	TN		HS				PF				P-value			
Growth^2^/Diet (D)^3^	C	SP	C			SP	C		SP		D		E	E x D
Hot carcass	73.4 ± 0.15	74.1 ± 0.19	72.9 ± 0.38			73.6 ± 0.35	74.0 ± 0.24		74.3 ± 0.40		**0.042**		**0.0243**	0.8298
Fat pad	0.80 ± 0.01	0.88 ± 0.02	0.95 ± 0.04			0.94 ± 0.04	0.78 ± 0.04		0.82 ± 0.02		0.1989		**0.0005**	0.4306
CWOG	75.1 ± 0.18	75.7 ± 0.26	74.4 ± 0.47			75.1 ± 0.39	75.7 ± 0.33		76.2 ± 0.48		0.0528		**0.0118**	0.9661
Breast	21.9 ± 0.21	22.7 ± 0.21	21.2 ± 0.22			21.9 ± 0.30	22.4 ± 0.18		22.3 ± 0.28		**0.0267**		**0.0037**	0.1402
Tender	3.7 ± 0.03^b^	3.8 ± 0.02^ab^	3.7 ± 0.02^b^			3.7 ± 0.03^b^	3.9 ± 0.02^a^		3.8 ± 0.03^ab^		0.1915		**0.0006**	**0.0258**
Wings	7.6 ± 0.08	7.6 ± 0.06	7.7 ± 0.08			7.6 ± 0.09	7.7 ± 0.07		7.7 ± 0.08		0.9645		0.6133	0.9158
Leg Quarter	22.4 ± 0.18	22.3 ± 0.26	22.7 ± 0.20			22.7 ± 0.14	22.4 ± 0.09		22.7 ± 0.19		0.6788		0.1279	0.4053
Main effect of diet														
	C				SP					P-value				
Hot carcass	73.5 ± 0.20				74.0 ± 0.21					0.086				
Breast	21.8 ± 0.19				22.3 ± 0.18^*^					**0.0494**				
Main effect of environment														
	TN			HS				PF				P-value		
Hot carcass	73.8 ± 0.16^ab^			73.3 ± 0.28^a^				74.2 ± 0.24^b^				**0.0234**		
Fat pad	0.84 ± 0.02^a^			0.95 ± 0.03^b^				0.80 ± 0.02^a^				**0.0001**		
CWOG	75.4 ± 0.20^ab^			74.7 ± 0.33^a^				76.0 ± 0.31^b^				**0.0058**		
Breast	22.3 ± 0.20^a^			21.5 ± 0.22^b^				22.4 ± 0.17^a^				**0.0002**		

^1^ HS, heat stress; PF, pair fed; TN, thermoneutral. ^2^CWOG, chilled carcass without giblet. ^3^C, control diet; SP, *Spirulina Plantensis.*Bold values indicate significant P values.

**Table 5:** Effect of *SP* on muscle myopathy incidences in heat-stressed broilers.

Environment (E)^1^	TN		HS		PF		P-value		
MM incidence^2^/Diet (D)^3^	C	SP	C	SP	C	SP	E	D	E x D
WS (%)									
NORM	15.0 ± 7.5	25.0 ± 8.3	32.5 ± 5.4	20.0 ± 6.1	22.5 ± 7.4	20.0 ± 7.9	0.6606	0.7794	0.3151
MOD	47.5 ± 4.1	40.0 ± 6.1	40.0 ± 7.1	55.0 ± 10.9	40.0 ± 7.1	42.5 ± 4.1	0.6706	0.5648	0.2939
SEV	37.5 ± 6.5	35.0 ± 5.6	27.5 ± 8.9	25.0 ± 5.6	37.5 ± 7.4	37.5 ± 4.1	0.197	0.7584	0.9759
WB (%)									
NORM	15.0 ± 2.5	30.0 ± 6.1	35.0 ± 7.5	22.5 ± 4.1	27.5 ± 4.1	22.5 ± 5.4	0.4985	0.8474	**0.0452**
MOD	32.5 ± 7.4	12.5 ± 4.1	50.0 ± 12.7	50.0 ± 7.9	25.0 ± 7.5	45.0 ± 7.5	**0.0132**	>0.9999	0.0791
SEV	52.5 ± 5.4	57.5 ± 8.2	15.0 ± 5.6	27.5 ± 7.4	47.5 ± 8.2	32.5 ± 8.9	**0.001**	0.892	0.1876
Main effect of environment									
	TN		HS		PF		P-value		
WB (%)									
MOD	22.50 ± 5.75^a^		50.00 ± 10.3^b^		35.00 ± 7.50^ab^		**0.05**		
SEV	55.00 ± 6.80^a^		21.25 ± 6.50^b^		40.00 ± 8.55^ab^		**0.0051**		

^1^ HS, heat stress; PF, pair fed; TN, thermoneutral. ^2^MM, muscle myopathy; MOD, moderate; NORM, normal; SEV, severe; WB, woody breast; WS, white striping.^3^C, control; SP, *spirulina plantens.*Bold values indicate significant P values.

### *In-feed SP* activates mTOR signaling pathway in breast muscle of heat-stressed broilers

**Figure 3:**
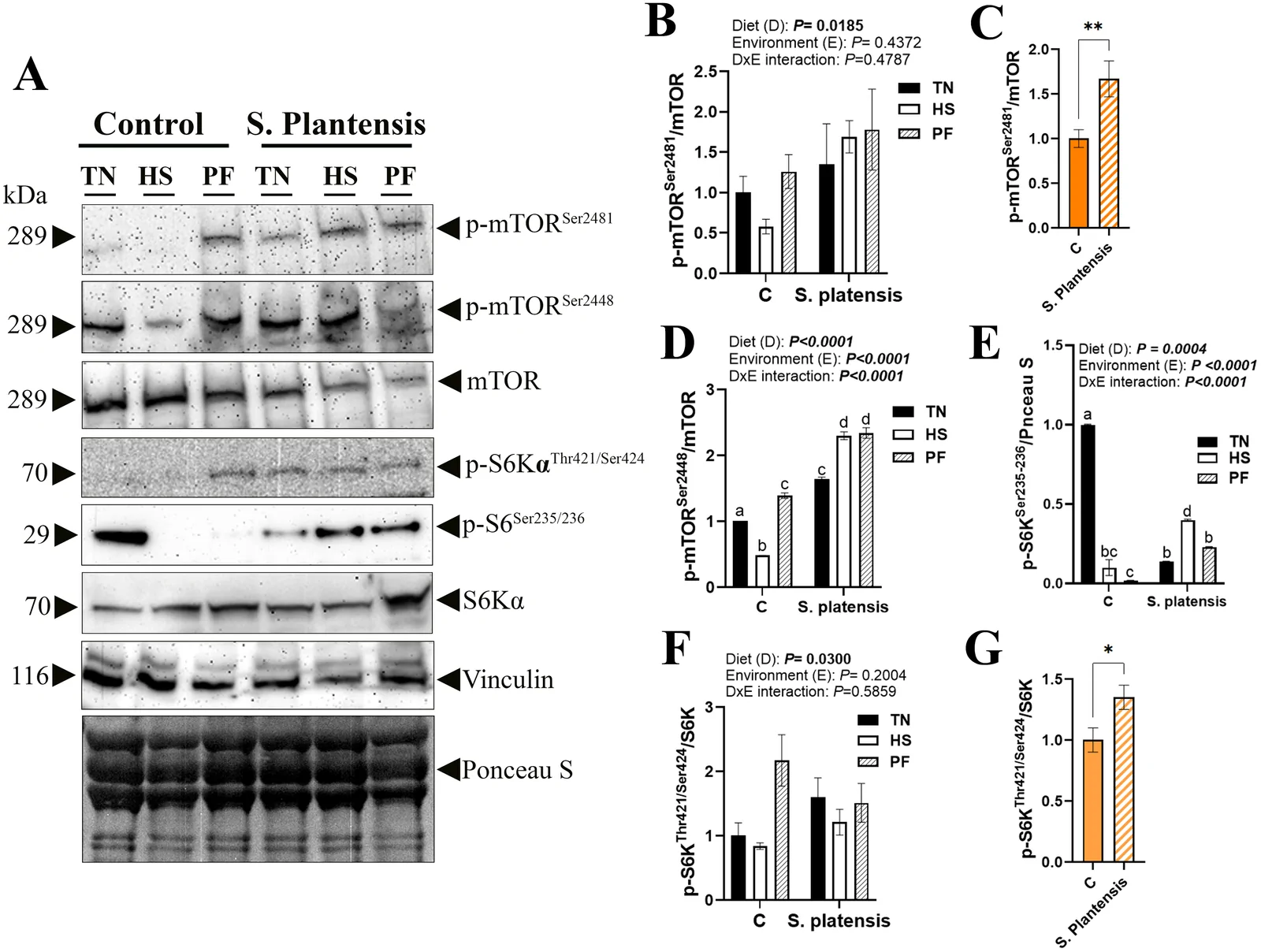
Effect of in-feed supplementation of *SP* on mTOR pathway in breast of heat-stressed broilers. Protein levels were determined by Western blot analysis **(A–G)** and the relative expression was represented as the ratio of target phosphorylated to pan protein. Data are presented as mean ± SD (n=6 birds/group) and analyzed by Two-way ANOVA and Tukey’s HSD multiple comparison test. If the diet by environment interaction is not significant, the main effect (D or E) was analyzed separately by Student T test or one-way ANOVA as appropriate. * and different superscript letters indicate significant difference at *P* < 0.05. The immunoblot is a representative illustration of 6 biological replicates. HS, heat stress; mTOR, mechanistic target of rapamycin; S6Kα, p70S6 kinase alpha; TN, thermoneutral; PF, pair fed.

Chronic exposure to HS decreased the phosphorylated levels of mTOR and S6K in birds fed control diet, but the differences were statistically discernable only for p-mTOR^Ser2448^ and p-S6^Ser235^/^236^ (**Figures 3A–G**). Supplementation of *SP* significantly increased the phosphorylated levels of mTOR at both ser2481 and ser2448 sites as well as S6K at thr421/ser424 and S6 at Ser235/236 sites compared to the control diet (**Figures 3A–G**). Pair feeding significantly upregulated the breast muscle expression of *mTOR*, *RPS6KB1*, and *RPS6KC1* genes compared to TN condition (**Supplementary Figures 1A, B, G–I, J**). mRNA abundances of *RPS6KA3*, *RPS6KA6*, and *EiF4EBP1* did not differ between TN, HS, and PF groups (**Supplementary Figures 1C–F, K, L**). The expression of breast *RPS6KA5* gene was not affected either by diet or environmental conditions (data not shown).

### *In-feed SP* deactivates eIF2α subunit in breast muscle of heat-stressed broilers

**Figure 4:**
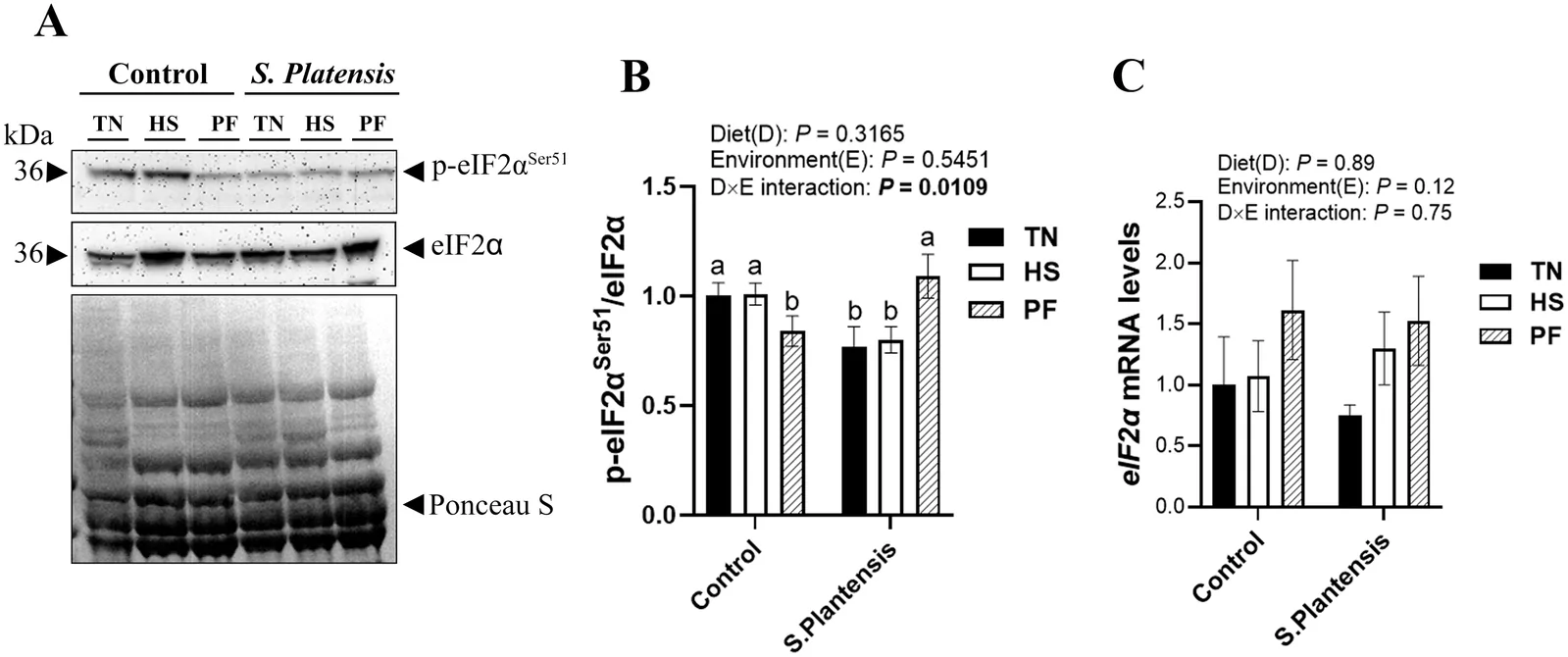
Effect of in-feed supplementation of *SP* on eIF2α in breast of heat-stressed broilers. The levels of eIF2α protein **(A, B)** and mRNA **(C)** were determined by Western blot and qPCR, respectively. Data are presented as mean ± SD (n=6 birds/group) and analyzed by Two-way ANOVA and Tukey’s HSD multiple comparison test. Different superscript letters indicate significant difference at *P* < 0.05. The immunoblot is a representative illustration of 6 biological replicates. eIF2α, eukaryotic translation initiation factor 2 alpha subunit; HS, heat stress; TN, thermoneutral; PF, pair fed.

Administration of *SP* significantly decreased p-eIF2α^Ser51^ levels in the breast muscle of broilers maintained under both TN and HS conditions (**Figures 4A, B**), but it increased it in the PF group (**Figures 4A, B**). The relative expression of eIF2α gene was not affected by diet or environmental conditions (**Figure 4C**).

### *In-feed SP* modulates ubiquitin-proteasome and endocytosis pathways in breast muscle of heat-stressed broilers

**Figure 5:**
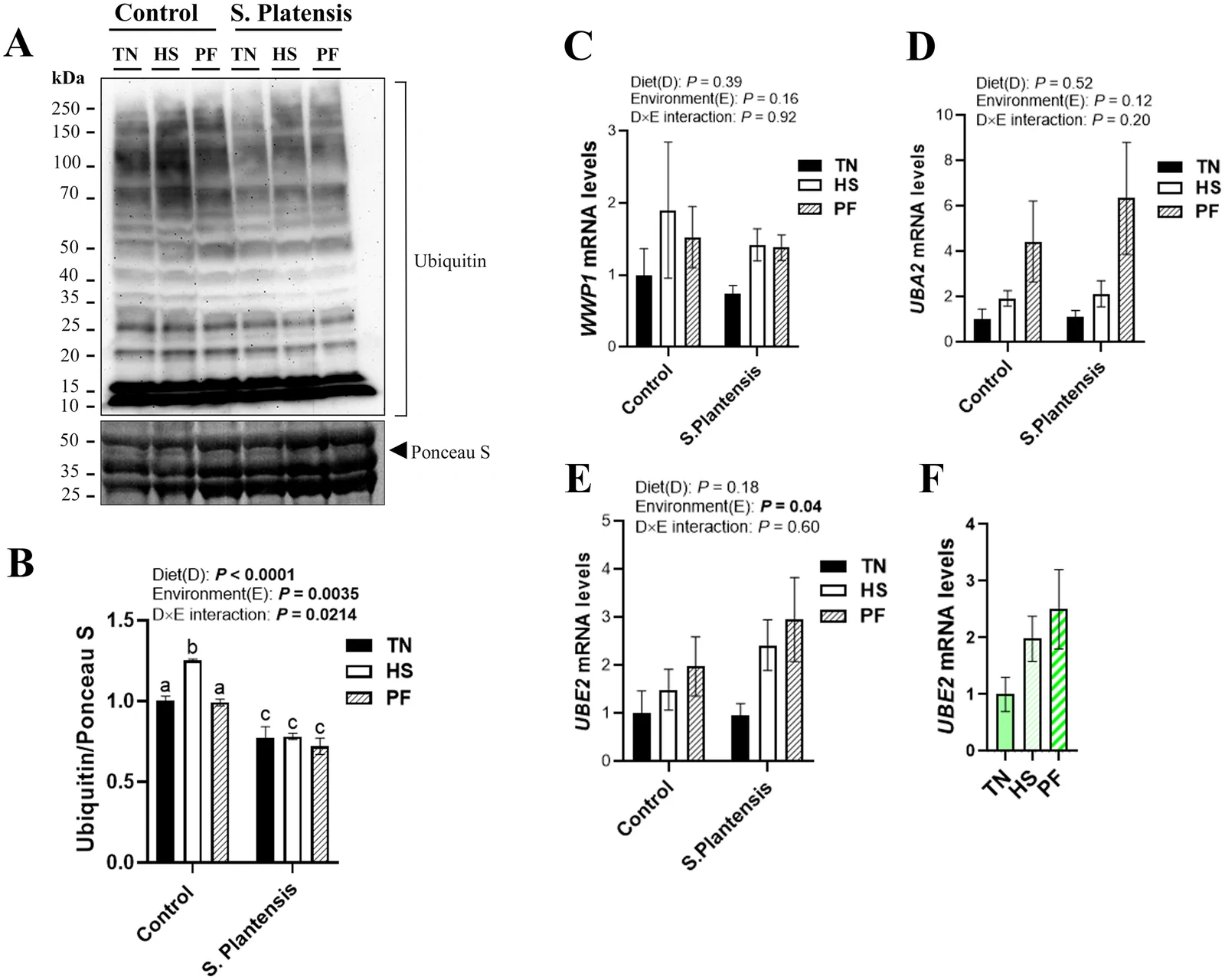
Effect of in-feed supplementation of *SP* on the expression of ubiquitin system in breast of heat-stressed broilers. The levels of ubiquitin protein **(A, B)** and mRNA of *WWP1* **(C)**, *UBA2* **(D)**, and *UBE2* **(E, F)** were determined by Western blot and qPCR, respectively. Data are presented as mean ± SD (n=6 birds/group) and analyzed by Two-way ANOVA and Tukey’s HSD multiple comparison test. If the diet by environment interaction is not significant, the main effect (D or E) was analyzed separately by Student T test or one-way ANOVA as appropriate. Different superscript letters indicate significant differences at *P* < 0.05. The immunoblot is a representative illustration of 6 biological replicates. HS, heat stress; PF, pair fed; TN, thermoneutral; UBA2, ubiquitin like modifier activating enzyme 2; UBE2, ubiquitin-conjugating enzymes E2; WWP1, WW domain containing E3 ubiquitin protein ligase.

**Figure 6:**
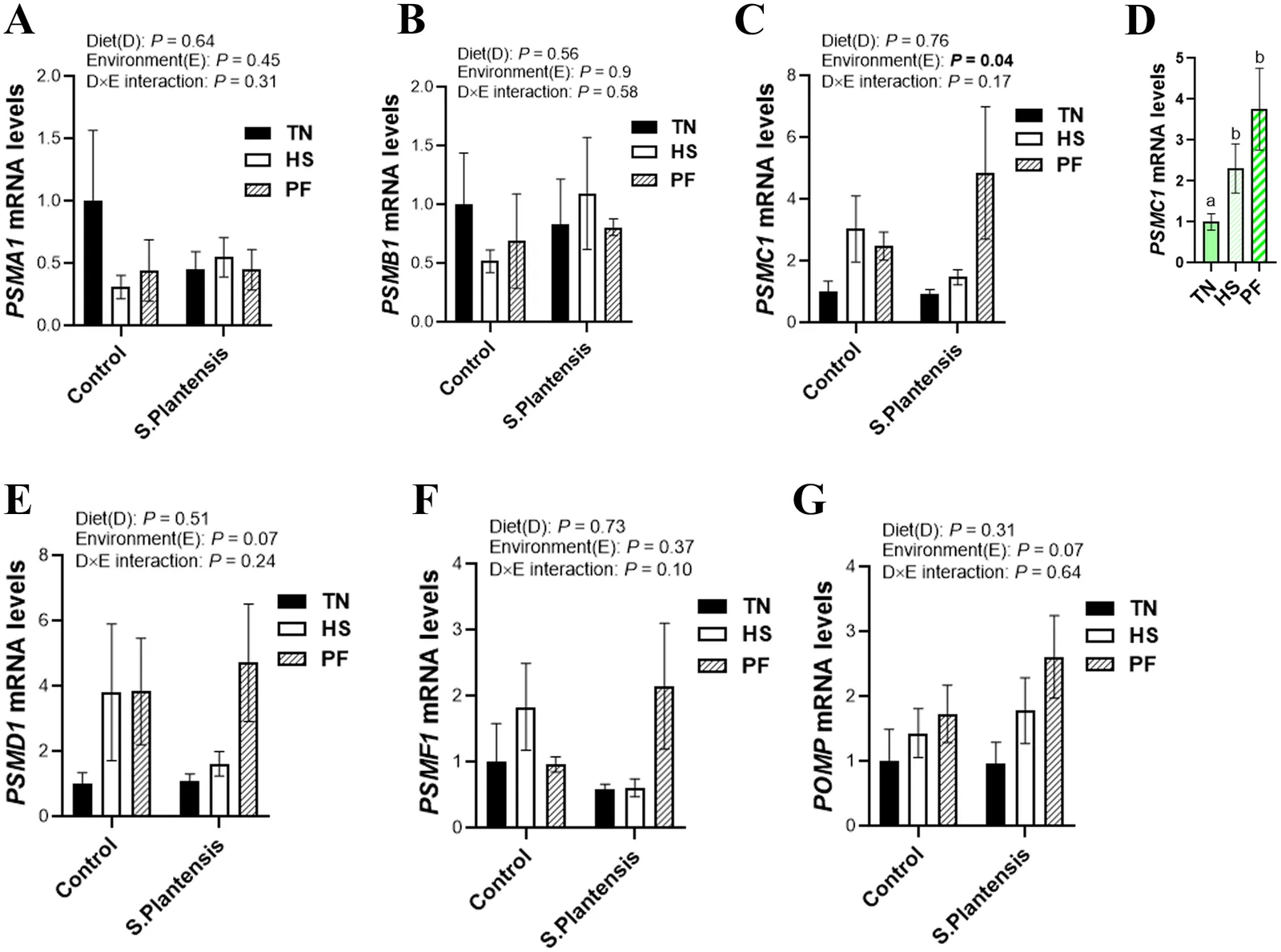
Effect of in-feed supplementation of *SP* on the expression of proteasome-associated genes in breast of heat-stressed broilers. Real-time qPCR was used to determine the gene expression of *PSMA1* **(A)**, *PSMB1* **(B)**, *PSMC1* **(C, D)**, *PSMD1* **(E)**, *PSMF1* **(F)**, and *POMP* **(G)**. Data are presented as mean ± SD (n=6 birds/group) and analyzed by Two-way ANOVA and Tukey’s HSD multiple comparison test. If the diet by environment interaction is not significant, the main effect (D or E) was analyzed separately by Student T test or one-way ANOVA as appropriate. Different superscript letters indicate significant differences at *P* < 0.05. HS, heat stress; PF, pair fed; *POMP*, proteasome maturation protein; *PSMA1*, proteasome subunit alpha 1; *PSMB1*, proteasome subunit beta 1; *PSMC1*, proteasome 26S subunit, ATPase 1; *PSMD1*, proteasome 26S subunit, non-ATPase 1; *PSMF1*, proteasome inhibitor subunit 1; TN, thermoneutral.

**Figure 7:**
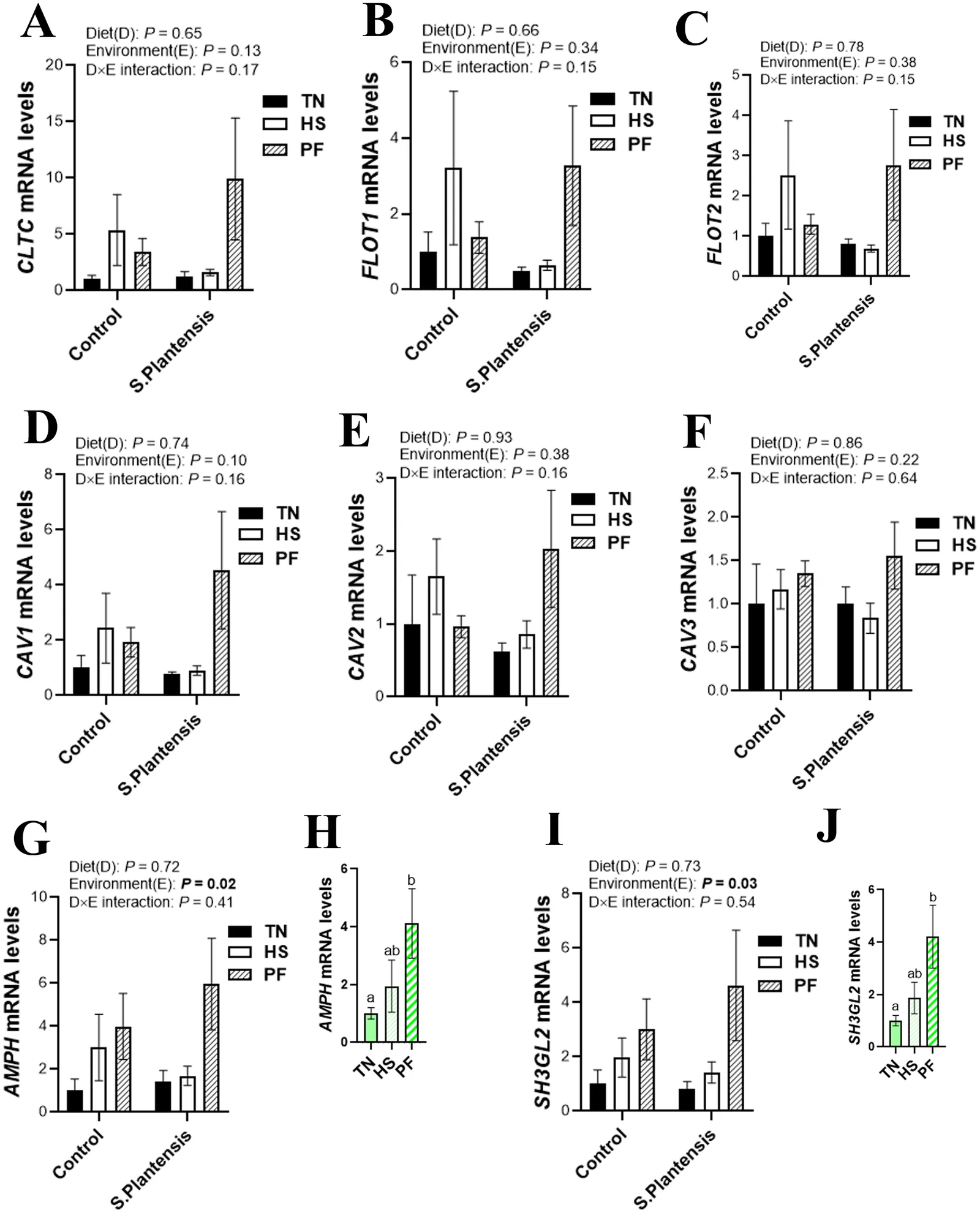
Effect of in-feed supplementation of *SP* on the expression of endocytosis machinery-associated genes in breast of heat-stressed broilers. Real-time qPCR was used to determine the gene expression of *CLTC* **(A)**, *FLOT1* **(B)**, *FLOT2* **(C)**, *CAV1* **(D)**, *CAV2* **(E)**, *CAV3* **(F)**, *AMPH* **(G, H)**, and *SH3GL2* **(I, J)**. Data are presented as mean ± SD (n=6 birds/group) and analyzed by Two-way ANOVA and Tukey’s HSD multiple comparison test. If the diet by environment interaction is not significant, the main effect (D or E) was analyzed separately by Student T test or one-way ANOVA as appropriate. Different superscript letters indicate significant differences at *P* < 0.05. *AMPH*, amphiphysin; *CAV*, caveolin; *CLTC*, Clathrin heavy chain; *FLOT*, flotillin; HS, heat stress; PF, pair fed; *SH3GL2*, SH3 domain containing GRB2 like 2; TN, thermoneutral.

As shown in **Figures 5A, B**, immunoblot analysis indicated that ubiquitin expression was elevated in heat-stressed birds compared to the TN group. Supplementing broilers with *SP* significantly decreased ubiquitin protein levels in all (TN, HS, and PF) groups (**Figures 5A, B**). The expression of breast WW domain containing E3 ubiquitin protein ligase 1 (*WWP1)*, ubiquitin like modifier activating enzyme 2 (*UBA2)*, and ubiquitin conjugating enzymes E2 (*UBE2)* remained unchanged between all tested groups (**Figures 5C–F**). Heat stress and pair-feeding significantly upregulated the breast expression of proteasome 26S subunit, ATPase (*PSMC1*, **Figures 6C, D**), but not of proteasome 20S subunit alpha 1 (*PSMA1)*, proteasome 20S subunit beta 1 (*PSMB1)*, proteasome 26S subunit, non-ATPase 1 (*PSMD1)*, proteasome inhibitor subunit 1 (*PSMF1)*, and proteasome maturation protein (*POMP)* (**Figures 6A, B, E-G**). Administration of *SP* did not affect the expression of any measured proteasome-related genes (**Figures 6A–G**). Heat stress did not affect the expression of endocytosis-related genes (clathrin heavy chain *CLTC*, Flotillin *FLOTs*, caveolin *CAVs*, amphiphysin *AMPH*, and SH3 domain containing GRB2 like 2 or endophilin-A1, *SH3GL2*) (**Figures 7A–J**), however pair feeding significantly upregulated the expression of breast *AMPH* and *SH3GL2* genes compared to TN conditions (**Figures 7G–J**).

### *In-feed SP* modulates the expression of heat shock proteins in breast muscle of heat-stressed broilers

**Figure 8:**
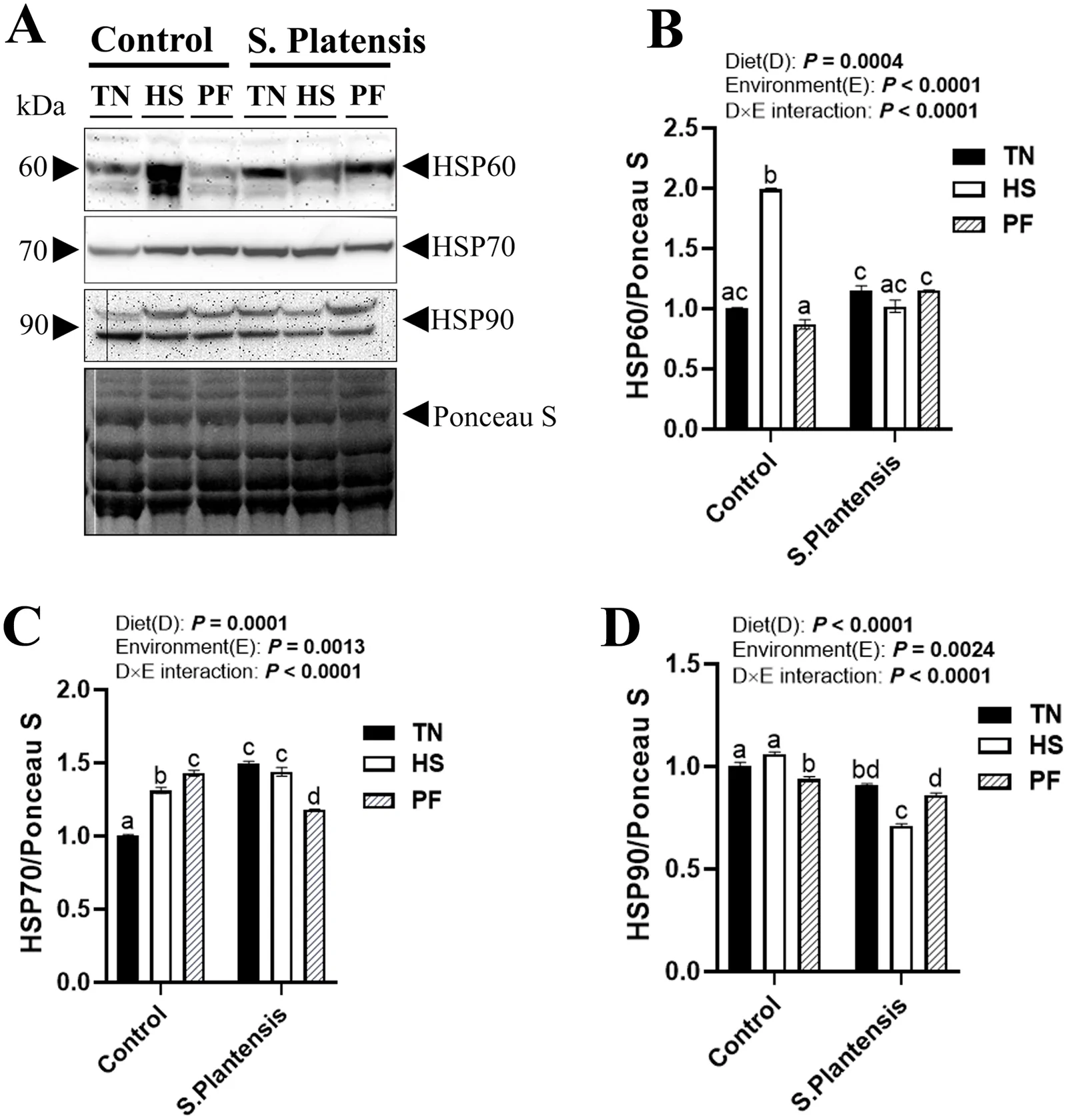
Effect of in-feed supplementation of *SP* on the expression of HSPs in breast of heat-stressed broilers. Western blot was used to determine protein levels of HSP60, HSP70, and HSP90 **(A)**. The relative expressions of HSP60 **(B)**, HSP70 **(C)**, and HSP90 **(D)** were presented as the ratio of the target HSP to Ponceau (S) Data are presented as mean ± SD (n=6 birds/group) and analyzed by Two-way ANOVA and Tukey’s HSD multiple comparison test. Different superscript letters indicate significant differences at *P* < 0.05. The immunoblot is a representative illustration of 6 biological replicates.

There was a significant environment by diet interactions for all measured HSPs (**Figures 8A–D**). Chronic HS significantly upregulated the expression of HSP60 and HSP70, but not that of HSP90 protein in birds fed with control diet (**Figures 8A–D**). Supplementation of *SP* significantly reduced the levels of HSP60 and HSP90 and increased that of HSP70 protein in heat-stressed broilers (**Figures 8A–D**). In the pair-fed group, *SP* administration significantly decreased the levels of HSP70 and HSP90 but increased that of HSP60 protein (**Figures 8A–D**).

### Treatments with QCT or BA partially reproduce *SP’s* effects in CPEM

**Figure 9:**
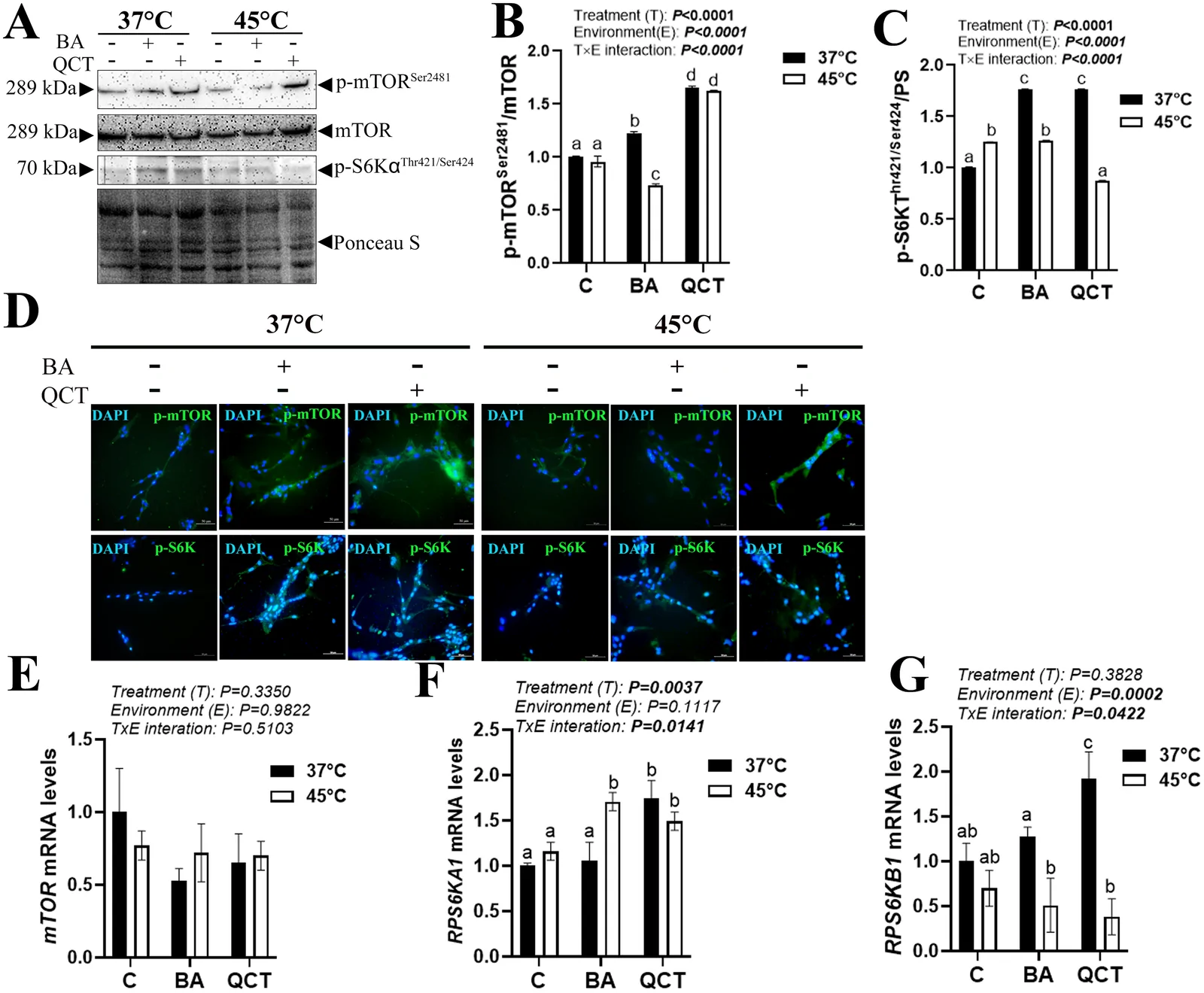
Effect of BA and QCT treatments on mTOR pathway in CPEM. Myoblasts were treated with BA (50 µM) or QCT (10 µM) and protein expression was determined by Western blot **(A-C)** and immunofluorescence **(D)**. Gene expressions of *mTOR* **(E)**, *RPS6KA1* **(F)**, and *RPS6KB1* **(G)** were determined by qPCR. Data are presented as mean ± SD (n=6 replicates/treatment) and analyzed by Two-way ANOVA and Tukey’s HSD multiple comparison test. Different superscript letters indicate significant differences at *P* < 0.05. The immunoblot is a representative illustration of 6 biological replicates. C, control; BA, benzoic acid, QCT, quercetin; mTOR, mechanistic target of rapamycin; *RPS6KA1*, ribosomal protein S6 kinase alpha-1; *RPS6KB1*, ribosomal Protein S6 Kinase Beta-1.

**Figure 10:**
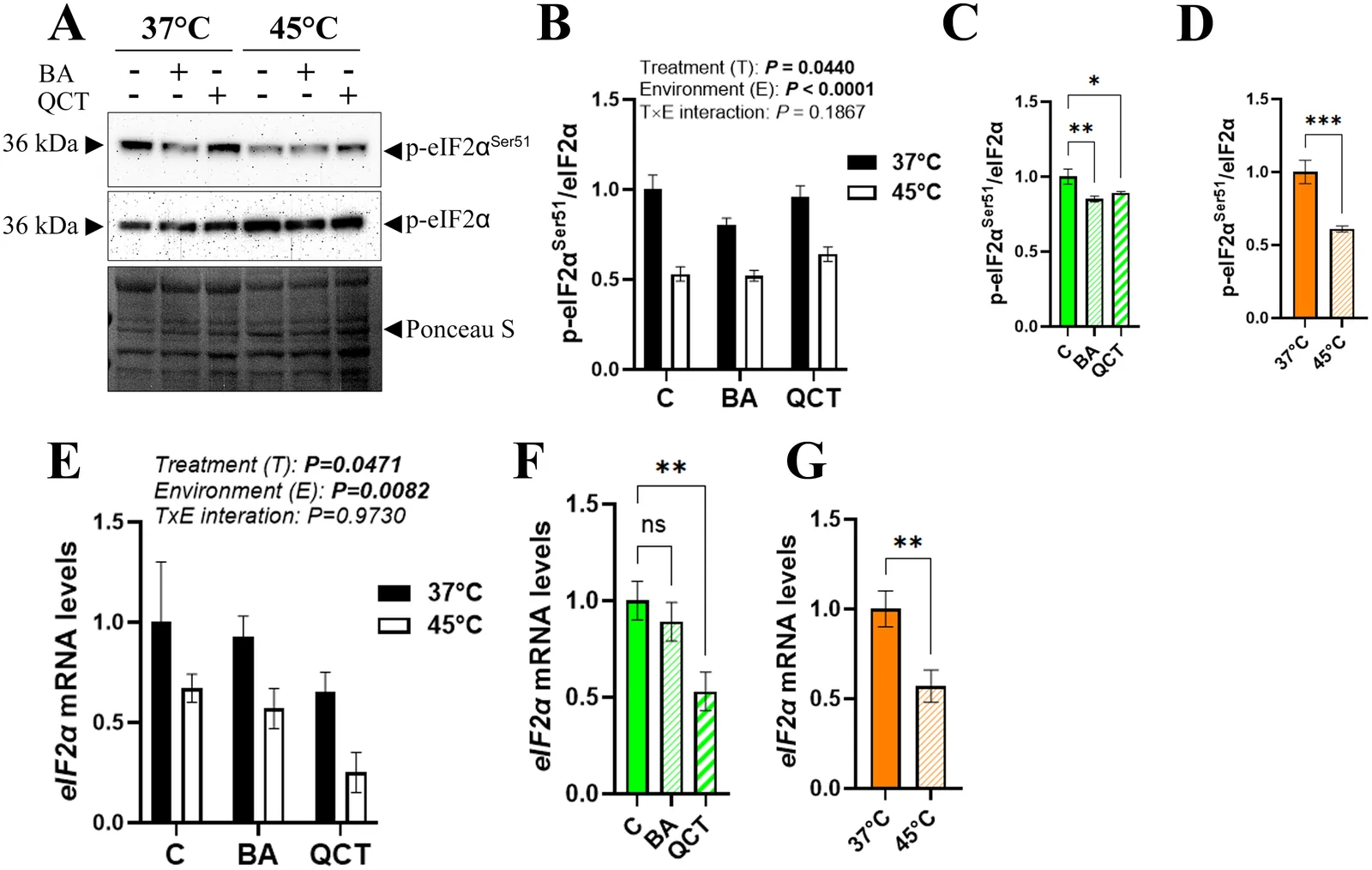
Effect of BA and QCT treatments on eIF2α pathway in CPEM. The levels of eIF2α protein **(A-D)** and mRNA **(E-G)** were determined by Western blot and qPCR, respectively. Data are presented as mean ± SD (n=6 replicates/treatment) and analyzed by Two-way ANOVA and Tukey’s HSD multiple comparison test. *, **, *** indicate significant differences at *P* < 0.05; *P* < 0.01; and *P* < 0.001, respectively. The immunoblot is a representative illustration of 6 biological replicates. C, control; BA, benzoic acid: eIF2α, eukaryotic translation initiation factor 2 alpha subunit; QCT, quercetin.

When the CPEM cells were maintained at 37°C, immunoblot analysis showed that treatments with BA or QCT significantly increased the phosphorylated levels of mTOR^Ser2481^ and S6K^Thr421/Ser424^ (**Figures 9A–C**). Under HS (45°C) conditions, QCT activated mTOR, but significantly decreased p-S6K^Thr421/Ser424^ levels, and BA significantly decreased p-mTOR^Ser248^ without affecting p-S6K^Thr421/Ser424^ (**Figures 9A–C**), which is in agreement with the immunofluorescence staining (**Figure 9D**). At mRNA levels, *mTOR* abundances remained unchanged (**Figure 9E**), however *RPS6KA1* expression was significantly upregulated and *RPS6KB1* was down regulated by both BA and QCT in heat-stressed cells (**Figures 9F, G**). The expressions of other *RPS6K* subunits (A3, A5, A6, B2, and C1) were not affected by treatments or HS (**Table 6**). Both phosphorylated levels of eIF2α at Ser51 site and its mRNA expression were significantly down regulated by HS and by BA or QCT treatment compared to 37°C or untreated cells, respectively (**Figures 10A–G**).

**Table 6:** Effect of BA and QCT on proteostasis-associated genes in heat-stressed chicken primary embryonic myoblasts.

Environment (E)	37°C			45°C			*P*-value		
Genes^1^/Treatment (T)^2^	C	BA	QCT	C	BA	QCT	E	T	E x T
Protein synthesis									
*RPS6KA3*	1.00 ± 0.20	0.66 ± 0.07	0.54 ± 0.06	0.83 ± 0.30	1.05 ± 0.30	0.90 ± 0.20	0.651	0.4683	0.2605
*RPS6KA5*	1.00 ± 0.30	1.16 ± 0.20	1.23 ± 0.40	1.08 ± 0.10	2.50 ± 0.80	1.68 ± 0.60	0.1183	0.2626	0.3988
*RPS6KA6*	1.00 ± 0.30	0.53 ± 0.04	0.51 ± 0.10	0.55 ± 0.10	0.89 ± 0.40	0.53 ± 0.30	0.9086	0.5686	0.2788
*RPS6KB2*	1.00 ± 0.30	0.60 ± 0.20	0.53 ± 0.30	1.30 ± 0.70	0.73 ± 0.20	0.70 ± 0.09	0.4995	0.2764	0.9693
*RPS6KC1*	1.00 ± 0.30	0.53 ± 0.40	0.19 ± 0.10	0.35 ± 0.30	0.58 ± 0.40	0.48 ± 0.20	0.5345	0.6809	0.2966
Proteasome									
*PSMD1*	1.00 ± 0.50	1.11 ± 0.20	1.40 ± 0.60	1.39 ± 0.20	1.99 ± 1.00	1.27 ± 0.50	0.4238	0.8223	0.6797
*PSME1*	1.00 ± 0.30	1.97 ± 1.00	1.68 ± 0.80	2.61 ± 1.00	1.26 ± 0.20	1.44 ± 0.80	0.7249	0.9437	0.2909
*PSMF1*	1.00 ± 0.20	1.20 ± 0.10	0.95 ± 0.10	1.00 ± 0.10	1.46 ± 0.40	0.98 ± 0.50	0.6805	0.3826	0.882
Endocytosis									
*AMPH*	1.00 ± 0.10	0.51 ± 0.08	0.69 ± 0.10	1.30 ± 0.40	1.05 ± 0.40	1.36 ± 0.70	0.1161	0.6099	0.8821
*CAV1*	1.00 ± 0.40	0.77 ± 0.10	0.74 ± 0.10	0.75 ± 0.10	0.51 ± 0.09	0.77 ± 0.20	0.3383	0.5115	0.7152
*CAV2*	1.00 ± 0.40	0.81 ± 0.10	0.56 ± 0.10	0.51 ± 0.10	0.63 ± 0.20	0.35 ± 0.20	0.1076	0.3253	0.7268
*CAV3*	1.00 ± 0.10	1.13 ± 0.10	0.97 ± 0.10	1.17 ± 0.10	1.27 ± 0.30	0.97 ± 0.50	0.6211	0.6648	0.9373
*CLTC*	1.00 ± 0.50	0.34 ± 0.10	0.20 ± 0.04	0.25 ± 0.07	0.41 ± 0.30	0.16 ± 0.10	0.2498	0.2237	0.2252
*FLOT1*	1.00 ± 0.10	1.48 ± 0.60	0.58 ± 0.10	2.71 ± 0.40	0.81 ± 0.40	1.51 ± 0.90	0.1263	0.2393	0.0795
*FLOT2*	1.00 ± 0.50	0.94 ± 0.20	0.54 ± 0.10	0.77 ± 0.30	0.64 ± 0.40	0.35 ± 0.20	0.38	0.3856	0.9856
*SH3GL2*	1.00 ± 0.50	0.22 ± 0.05	0.26 ± 0.09	0.44 ± 0.20	0.32 ± 0.20	0.45 ± 0.30	0.7072	0.2793	0.3857

^1^ AMPH, amphiphysin; CAV, caveolin; CLTC, CLTC, clathrin heavy chain; FLOT, flotillin; PSMD1, proteasome 26S subunit, non-ATPase; PSME1, proteasome activator subunit 1; PSMF1, proteasome inhibitor subunit 1; RPS6KA, ribosomal protein S6 kinase alpha; RPS6KB2, ribosomal protein S6 kinase B2.

^2^ C, control; BA, benzoic acid; QCT, quercetin.

**Figure 11:**
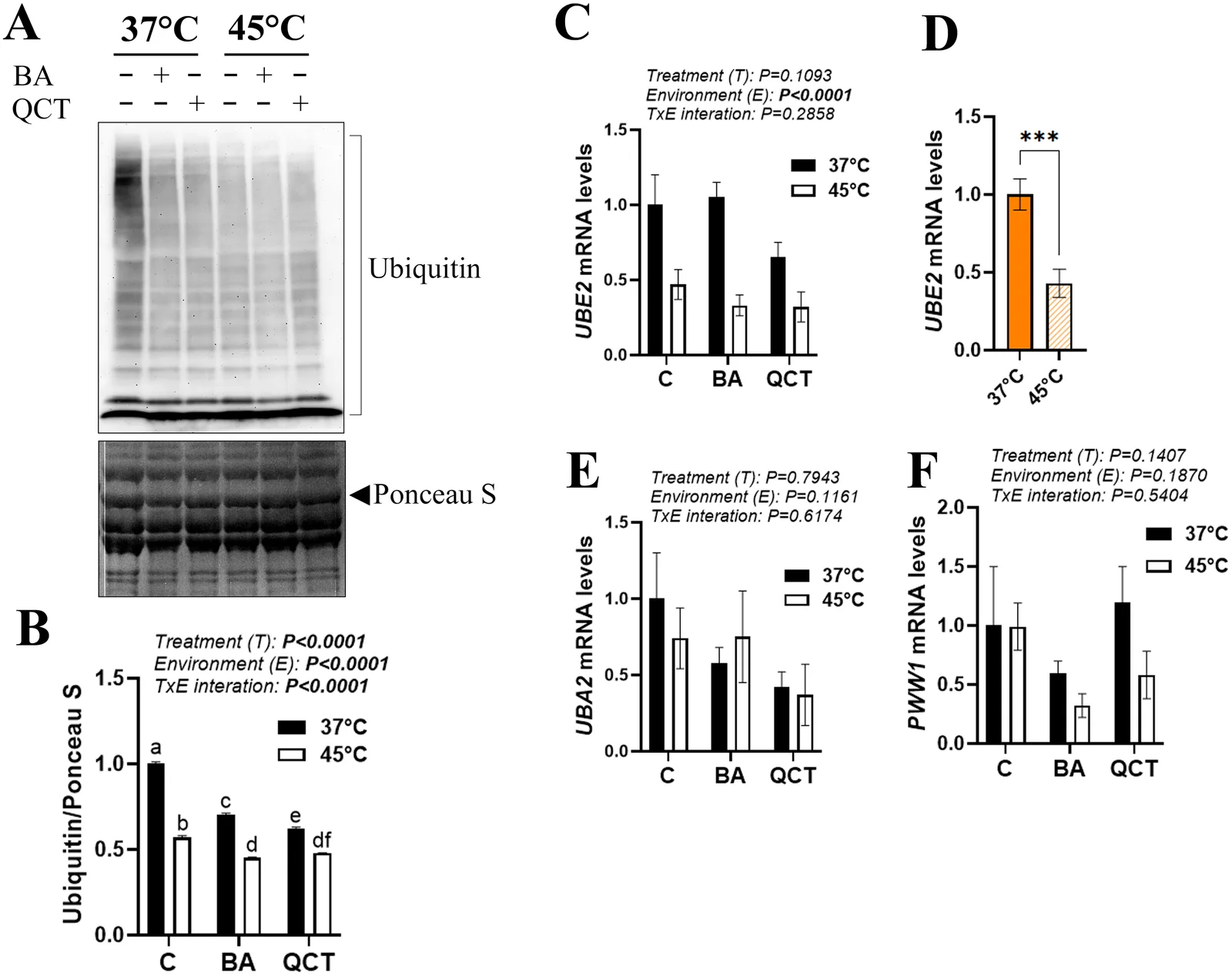
Effect of BA and QCT treatments on ubiquitin system in CPEM. The levels of ubiquitin protein **(A, B)** and mRNA of *UBE2* **(C, D)**, *UBA2* **(E)**, *WWP1* **(F)** were determined by Western blot and qPCR, respectively. Data are presented as mean ± SD (n=6 replicates/group) and analyzed by Two-way ANOVA and Tukey’s HSD multiple comparison test. If the treatment by environment interaction is not significant, the main effect (T or E) was analyzed separately by Student T test or one-way ANOVA as appropriate. *** and different superscript letters indicate significant differences at *P* < 0.001 and *P* < 0.05, respectively. The immunoblot is a representative illustration of 6 biological replicates. C, control; BA, benzoic acid; QCT, quercetin; *UBA2*, ubiquitin like modifier activating enzyme 2; *UBE2*, ubiquitin-conjugating enzymes E2; *WWP1*, WW domain containing E3 ubiquitin protein ligase.

**Figure 12:**
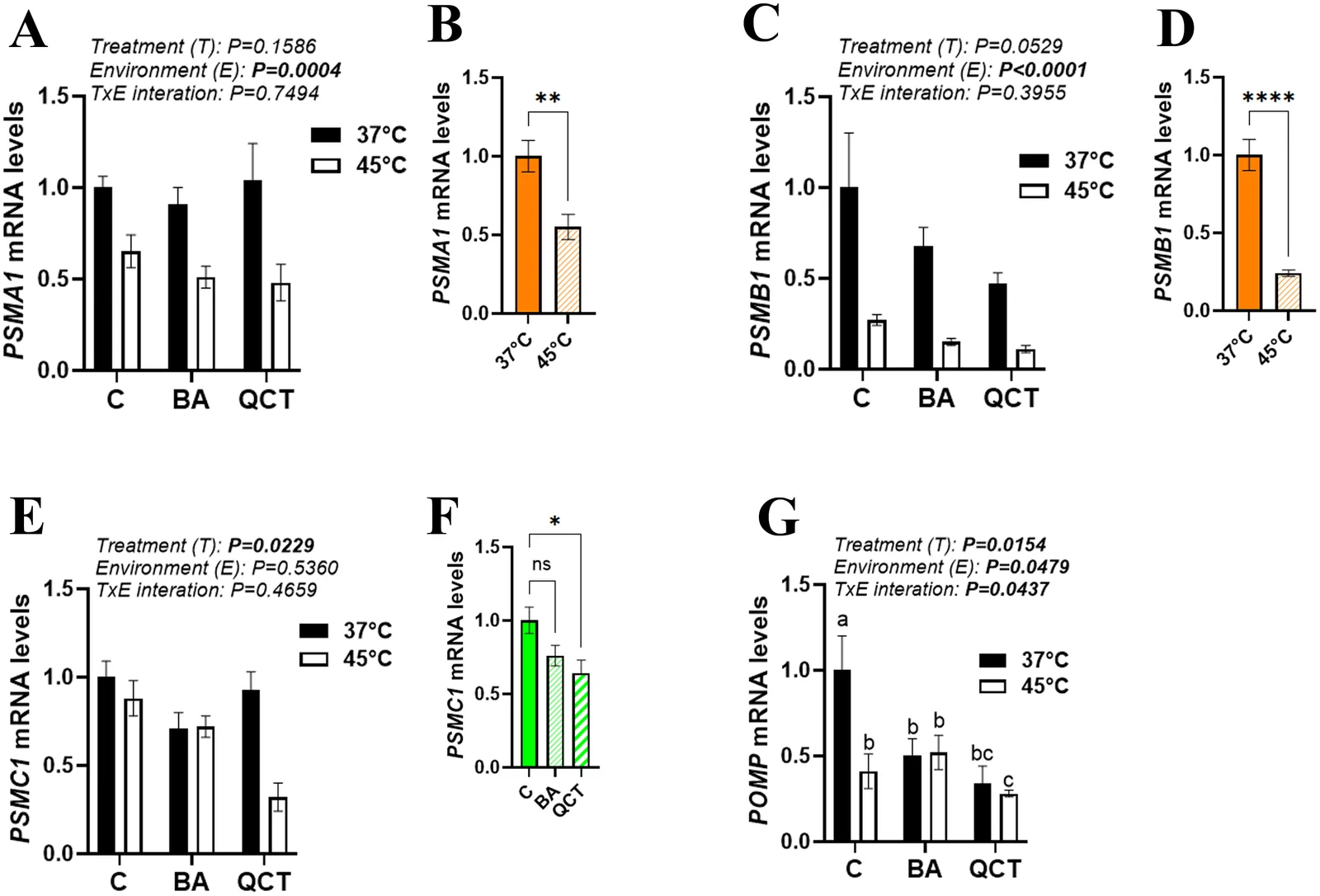
Effect of BA and QCT treatments on proteasome-associated genes in CPEM. Real-time qPCR was used to determine the gene expression of *PSMA1* **(A, B)**, *PSMB1* **(C, D)**, *PSMC1* **(E, F)**, and *POMP* **(G)**. Data are presented as mean ± SD (n=6 replicates/treatment) and analyzed by Two-way ANOVA and Tukey’s HSD multiple comparison test. If the treatment by environment interaction is not significant, the main effect (T or E) was analyzed separately by Student T test or one-way ANOVA as appropriate. *, **, **** indicate significant differences at *P* < 0.05; *P* < 0.001; and *P* < 0.0001, respectively. Different superscript letters indicate significant differences at *P* < 0.05. C, control; BA, benzoic acid; QCT, quercetin; *POMP*, proteasome maturation protein; *PSMA1*, proteasome subunit alpha 1; *PSMB1*, proteasome subunit beta 1; *PSMC1*, proteasome 26S subunit, ATPase 1.

Ubiquitin protein expression levels, detected by immunoblot analysis, were significantly reduced by HS and by treatments (BA and QCT) (**Figures 11A, B**). Gene expression of *UBE2* was significantly down regulated by HS (**Figures 11C, D**), however *UBA2* and *PWW1* mRNA levels were not affected by either HS or treatments (**Figures 11E, F**). The analysis of the proteasome complex showed that the subunits *PSMA1* and *PSMB1* expressions were significantly down regulated by HS (**Figures 12A–D**). The expression of *PSMC1* was decreased by both treatment BA and QCT, but the difference was statistically discernable only for QCT (**Figures 12E, F**). The expression of *POMP* was significantly down regulated by BA and QCT under 37°C, and only by QCT under 45°C, which resulted in a significant environment by treatment interaction (**Figure 11G**). The expressions of *PSMD1*, *PSME1*, and *PSMF1* genes were not affected by HS or treatments (**Table 6**).

The expression of genes involved in the endocytosis machinery, including amphiphysin (AMPH), caveolins (CAV1, CAV2, CAV3), clathrin heavy chain (CLTC), flotillins (FLOT1 and FLOT2), and SH3 domain containing GRB2 like 2 or endophilin A1 (SH3GL2) were not affected by either HS or treatments (**Table 6**).

**Figure 13:**
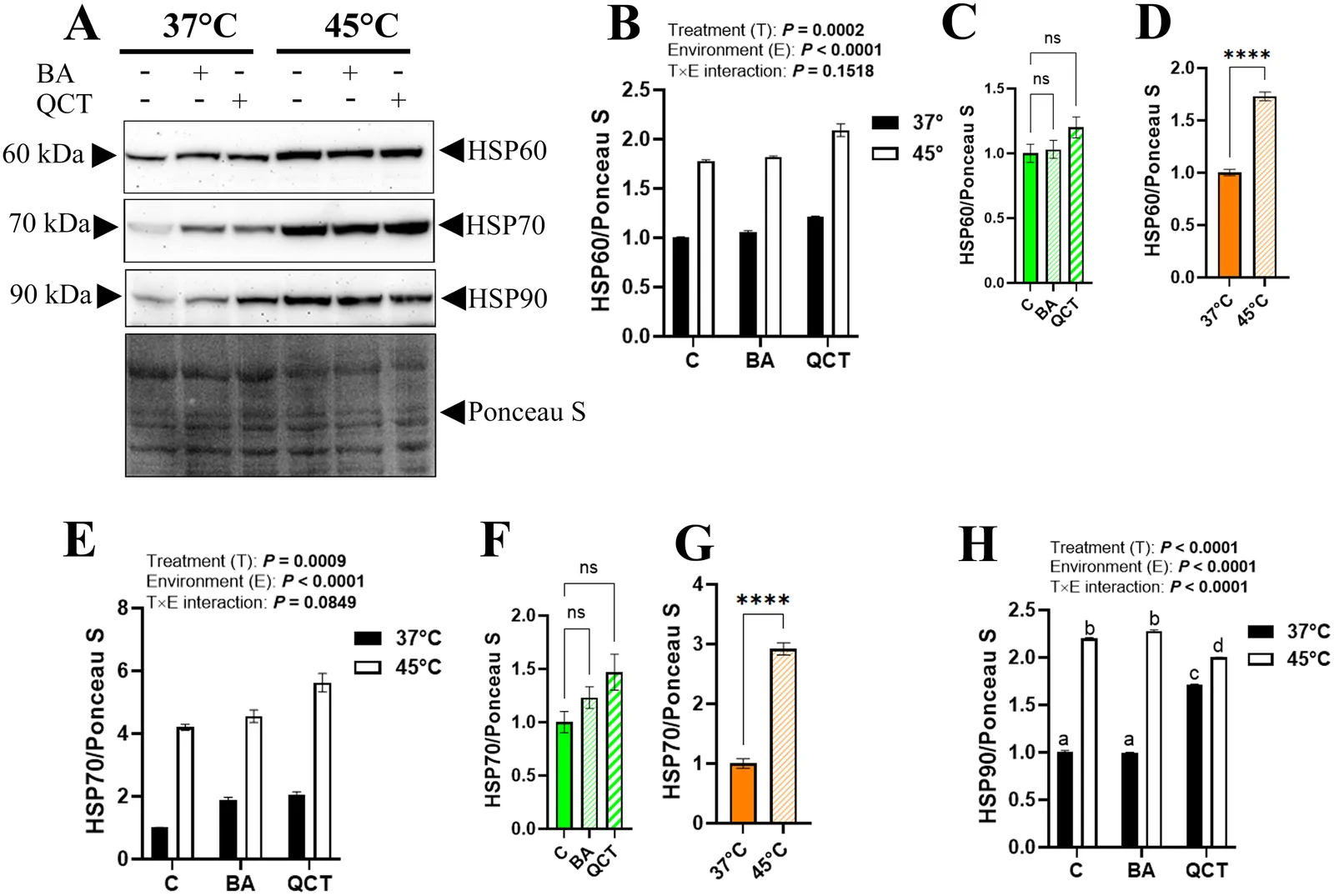
Effect of BA and QCT treatments on HSPs in CPEM. Western blot was used to determine protein levels of HSP60, HSP70, and HSP90 **(A)**. The relative expressions of HSP60 **(B-D)**, HSP70 **(E-G)**, and HSP90 **(H)** were presented as the ratio of the target HSP to Ponceau (S) Data are presented as mean ± SD (n=6 replicates/treatment) and analyzed by Two-way ANOVA and Tukey’s HSD multiple comparison test. **** and different superscript letters indicate significant differences at *P* < 0.0001 and *P* < 0.05, respectively. The immunoblot is a representative illustration of 6 biological replicates. C, control; BA, benzoic acid; HSP, heat shock protein; QCT, quercetin.

The regulation of HSPs in CPEM cells is summarized in **Figure 13**. Exposure to HS significantly increased the protein levels of HSP60, HSP70, and HSP90 (**Figures 13A-H**). Administration of BA or QCT numerically increased the expression of HSP60 and HSP70 in CPEM cells, but the differences were not statistically discernable at 0.05 level. Protein levels of HSP90, however, were affected only by QCT, where they were induced at 37°C and decreased at 45°C as compared to untreated cells (**Figure 13H**).

## Discussion

Undoubtedly heat load, aggravated by global warming (Steel et al., 2022), is a significant ongoing challenge to poultry production sustainability, as it impairs bird physiology, performances and welfare, and subsequently causes significant economic losses (Abbas et al., 2025). Among the promising nutritional strategies to mitigate the adverse effects of HS is the supplementation of microalgae that gained increasing attention in recent years as a sustainable and nutritious ingredient for animal feed (El-Shall et al., 2023), however its underlying mode of action is still not fully defined. Here, in-feed supplementation of the blue-green microalgae *SP* improved WCR (water intake: BW gain) by 36.5 points in heat-stressed broilers. Although the underpinning mechanisms warrant further investigations, it is possible that the WCR improvement was associated with decreased CBT following microalgae administration in heat-stressed birds. It is well known that heat-stressed birds lose water via panting, and according to the National Research Council, water consumption increases by roughly 7% for every 1°C increase in temperature over 21°C (National Research Council, 1994) to compensate for water loss and to enhance heat dissipation.

As anticipated, HS reduced growth performance, which corroborates previous studies, including our owns (Emami et al., 2021; Greene et al., 2021b; Aloui et al., 2024; Kim et al., 2025; Pontalti et al., 2025). Of particular interest, *SP* supplementation enhanced breast yield without affecting the incidence of muscle myopathies (WB and WS). It is worth highlighting that several published papers have reported inconsistent results, with some studies showing improvement of breast weight (Abbass et al., 2020; Abdelfatah et al., 2024), however others indicating a decrease or no change (Fathi et al., 2018; Park et al., 2018; Mishra et al., 2023). These discrepancies could be associated with various factors, including the experimental design (broiler strains, gender, age, trial duration, basal diet, housing and environmental conditions, production system, etc.) as well as *SP’*s source, origin, composition, form, administration route, storage conditions, and inclusion rate. The beneficial effects of *SP* on growth performances were reported to be linked to its ability to improve the immune system (Al-Batshan et al., 2001; Katayama et al., 2016; Fathi et al., 2018; El-Shall et al., 2023; Abdelfatah et al., 2024), modulate gut microbiota and increased count of beneficial lactic acid bacteria (Joya et al., 2021; Attia et al., 2023; Khalilnia et al., 2023; Alghamdi et al., 2024), enhance the antioxidant status and reduce the oxidative damage (Mirzaie et al., 2018; Hajati H et al., 2021).

Here, we provide new insights for the first time, to the best of our knowledge, regarding the mode of action of *SP*, where it modulates the broiler breast muscle proteostasis network. Proteostasis, coined by Balch and colleagues in February 2008 (Balch et al., 2008), refers to the cellular processes that maintain the integrity and quality of functional proteins and comprises three pathways: synthesis, folding, and degradation to govern protein homeostasis. The increased levels of phosphorylated mTOR at Ser2481 and Ser2448 sites, S6K^Thr421/Ser424^ and S6^Ser235/236^ support enhanced protein synthesis by *SP*. It is well known that mTOR is a central regulatory protein (Simcox and Lamming, 2022). When it is activated by phosphorylation, it initiates downstream signaling pathways, including S6K, to promote mRNA biogenesis, translation initiation and elongation, and thereby synthesis of new proteins (Laplante and Sabatini, 2012). This process is essential for cell growth, proliferation, and muscle hypertrophy (Laplante and Sabatini, 2012).

Enthrallingly, supplementation of *SP* decreased also the levels of phosphorylated eIF2α at Ser51 site in the breast muscle of heat-stressed birds, which indicates an additional potential mechanism for *SP* to enhance protein synthesis, possibly to adapt to stress induced by heat load. It is established that during cellular stress, translation initiation is downregulated by a mechanism involving the phosphorylation of eIF2α-subunit at Ser 51 (Adomavicius et al., 2019). The eIF2 forms an active ternary (translation initiation) complex with the translation initiator Met-tRNA and guanosine diphosphate (GDP), which is recruited to the 40S ribosome subunit along with other factors, such as eIF1, eIF3, and eIF5, to form the 43S preinitiation complex (43S PIC) (Asano et al., 2000; Shin et al., 2011). Phosphorylation of eIF2α^Ser51^ increases the binding of eIF2 to its guanine nucleotide exchange factor (GEF or eIF2B), resulting in sequestration and locks up that blocks the exchange activity of eIF2-GDP to eIF2-GTP and consequently inhibits translation and shuts down global protein synthesis (Krishnamoorthy et al., 2001; Jiang and Wek, 2005).

The other side of the coin for proteostasis network is protein degradation, which is controlled by the ubiquitin-proteasome system (UPS) (Bett, 2016). Ubiquitin is crucial for protein homeostasis and quality control by tagging misfolded, damaged, or short-lived proteins with chains for destruction by the proteasome (Pohl and Dikic, 2019; Pla-Prats and Thoma, 2022). Unsurprisingly, HS in our experimental conditions increased ubiquitin expression, which corroborates previous studies (Chung et al., 2000; Zhao et al., 2016; Lee and Goldberg, 2022). This indicates that HS hijacks the ubiquitin system to tag the stress-misfolded, -aggregated, and/or -damaged proteins for removal (Parag et al., 1987). It is also possible, according to a recent discovery, that HS-induced ubiquitination primes cells to dismantle stress granules and reinitiate normal cellular activities (Maxwell et al., 2021). Supplementation of *SP* decreased ubiquitin expression, indicating lower protein degradation (Hochstrasser, 1996), which in combination with higher protein synthesis as illustrated above might result in higher protein turnover and net muscle tissue building (Zhang et al., 2014). Proteasomes, large, multi-subunit protein complexes pivotal for degrading unwanted proteins, are essential for cellular health and homeostasis (Rousseau and Bertolotti, 2018). Here, the expressions of proteasome subunits *PSMA1*, *PSMB1*, *PSMD1*, *PSMF1*, and *POMP* genes were not affected by both HS and *SP* supplementation, however *PSMC1* mRNA abundances were upregulated in HS and PF groups. In combination with downregulated ubiquitin expression, this may suggest that *SP* reduced intracellular unwanted proteins, however duly noted, due to lack of proteasome subunit antibodies that cross-react with chickens, only mRNA levels were measured here. It is highly plausible that protein and mRNA levels are not correlated, a discrepancy that raises key questions and warrants future investigations.

Endocytosis is a key process for maintaining cellular plasma membrane proteostasis (Paul et al., 2023), by engulfing and removing damaged or aggregated cell surface proteins and delivering them for lysosomal degradation (Mukherjee et al., 1997; Sigismund et al., 2021). Here, endocytosis-related genes *CLTC*, *FLOTs*, and *CAVs* were not affected, however *AMPH* and *SH3GL2* were significantly upregulated by PF but not HS. Studies have shown that cellular stress induced by dietary restriction, indeed, induces endocytosis (Rainero et al., 2015; Muranen et al., 2017). Supplementation of *SP* did not affect endocytosis machinery, suggesting low levels or absent of damaged- and aggregated-surface proteins however take on board that only mRNA abundances were measured in our experimental conditions, and further in-depth investigations and analyses are needed.

Chaperonins are central to proteostasis by assisting protein folding, preventing protein aggregation, and directing misfolded proteins for degradation or refolding, ensuring cellular protein quality control particularly under stress (Kim et al., 2013). As expected, HS induced HSP60 and HSP70 protein levels in our experimental conditions, corroborating previous studies, including our owns (Leandro et al., 2004; Emami et al., 2021). Interestingly, feed restriction (pair-feeding) induced HSP70, reduced HSP90, and did not affect HSP60 protein levels compared to TN groups. This indicates that the effect of HS per se is different from that of feed depression-induced by heat load. Supplementation of *SP* decreased the protein levels of HSP60 and HSP90 but induced that of HSP70 in the breast of heat-stressed broilers. This suggests that HSPs may have different functions (holdases *vs.* foldases, assembly *vs*. disassembly, etc.) in chicken breasts or distinct roles in recognition of protein folding/refolding (Freeman and Morimoto, 1996). It is also possible that various active compounds of *SP* differentially trigger heat shock transcription factors, leading to varied expression levels and probably localizations of HSPs. Regardless of the specialized roles of HSPs in chicken breast, *SP* seems to modulate proteostasis network that merits deeper investigation.

Microalgae have been generally defined as a superfood due to their exceptional nutritional profile packed with diverse macro- and micro-nutrients (Podgorska-Kryszczuk, 2024). In attempt to mechanistically define the mode of action of *SP* and support the above-described *in vivo* data, we next determined the effects of BA and QCT, two dominant polyphenols quantified by LC-MS/MS analysis, on proteostasis in CPEM cells. Individual administration of BA or QCT partly recapitulated the effect of *SP*. For instance, QCT activated mTOR^Ser2481^but decreased p-S6K^Thr421/Ser24^. The mTOR activation observed here corroborates previous study in C2C12 cells (Hour et al., 2022), however it contradicts several data in cancer cells (Jia et al., 2018), which indicates that the effect of QCT depends on the (patho)physiological status of the cell/tissue. The upregulation of *RPS6KA1* gene suggests that QCT might target and activate specific p70S6K subunits downstream mTOR pathway. Moreover, QCT treatment decreased eIF2α at both mRNA and protein levels, ubiquitin protein expression, proteasome *PSMC1* mRNA abundances, and modulated chaperonin levels by reducing HSP90 expression. Together these data showed that QCT, as for *SP*, modulates proteostasis in CPEM by enhancing protein synthesis, reducing protein degradation, and modulating chaperonin expression. Administration of BA has similar effects as QCT, except for mTOR where there was a significant reduction of p-mTOR^Ser2481^ in heat-stressed CPEM. This suggests that BA might directly induce *RPS6KA1* gene transcription or activate a different upstream pathway, such as mitogen-activated protein kinase (Lin et al., 2007), adenosine monophosphate-activated protein kinase (Lee et al., 2014), protein kinase B or AKT (Lee and Lee, 2021), or others, all of which are known to regulate proteastosis (Garza-Lombo et al., 2018; Summers et al., 2018; Sun et al., 2025).

In summary, this is the first report to the best of our knowledge showing that in-feed supplementation of *SP* at 0.5% inclusion rate modulated proteostasis network through activation of mTOR-eIF2α pathway, reduction of ubiquitin protein expression, and regulation of HSPs, which in turn resulted in enhanced breast yield in heat-stressed broilers. The beneficial effects of *SP* seem to be associated with its active polyphenolic compounds (BA and QCT), however further studies are needed to evaluate other compounds individually or in combination.

## Data Availability

The raw data supporting the conclusions of this article will be made available by the authors, without undue reservation.

## Supplementary material

Supplementary Figure 1 Effect of in-feed supplementation of *SP* on gene expression associated with mTOR pathway. Real-time qPCR was used to determine the expression of *mTOR* (A, B), *RPS6KA3* (C, D), *RPS6KA6* (E, F), *RPS6KB1* (G, H), *RPS6KC1* (I, J), and *EiF4EBP1* (K, L). Data are presented as mean ± SD (n=6 birds/group) and analyzed by Two-way ANOVA and Tukey’s HSD multiple comparison test. Different superscript letters indicate significant differences at *P* < 0.05. EiF4EBP1, eukaryotic translation initiation factor 4E binding protein 1; mTOR, mechanistic target of rapamycin; RPS6KA, ribosomal protein S6 kinase alpha; RPS6KB, ribosomal protein S6 kinase beta; RPS6KC, ribosomal protein S6 kinase delta;

## Glossary

ADG: average daily gain
AMPH: amphiphysin
BA: benzoic acid
BW: body weight
C: control
CAV: caveolin
CBT: core body temperature
CFI: cumulative feed intake
CLTC: Clathrin heavy chain
CPEM: chicken primary embryonic myoblast
CWC: cumulative water consumption
DPPH: 2,2-diphenyl-1-picrylhydrazyl
EiF2α: eukaryotic translation initiation factor 2 alpha
EiF4EBP1: eukaryotic translation initiation factor 4E binding protein 1
FCR: feed conversion ratio
FLOT: flotillin
HS: heat stress
MOD: moderate
mTOR: mechanistic target of rapamycin
NORM: normal
POMP: proteasome maturation protein
PSMA1: proteasome 20S subunit alpha 1
PSMB1: proteasome 20S subunit beta 1
PSMC1: proteasome 26S subunit, ATPase 1
PSMD1: proteasome 26S subunit, non-ATPase 1
PSMF1: proteasome inhibitor subunit 1
QCT: quercetin
r18S: ribosomal 18S
RPS6KA: ribosomal protein S6 kinase alpha
RPS6KB: ribosomal protein S6 kinase beta
RPS6KC1: ribosomal protein S6 kinase delta-1
SEV: severe
SP: spirulina platensis
TN: thermoneutral
UBA2: ubiquitin like modifier activating enzyme 2
UBE2: ubiquitin conjugating enzyme E2
WB: woody breast
WCR: water conversion ratio
WS: white striping
WWP1: WW domain containing E3 ubiquitin protein ligase 1.
