# Supplementary material for: In-feed Arthrospira (Spirulina) platensis supplementation enhances breast yield in heat-stressed broilers through modulation of proteostasis network
Source: Front Physiol. 2026 Sep 9;17:1912121. doi: 10.3389/fphys.2026.1912121 (PMC13597348; doi:10.3389/fphys.2026.1912121)
Supplement: Supplementary file 1 [file Table1.docx]

**Supplementary Table 1**: Ingredient and nutrient composition of experimental starter, grower, and finisher diets

|  | Starter  (0-14d) | | Grower  (15-28d) | | Finisher  (29-42d) | |
| --- | --- | --- | --- | --- | --- | --- |
|  | Cont | Algae | Cont | Algae | Cont | Algae |
| Ingredient, % as-is |  |  |  |  |  |  |
| Corn | 55.1 | 55.1 | 60.3 | 60.3 | 63.7 | 63.7 |
| Soybean meal | 38.6 | 38.6 | 33.23 | 33.23 | 29.2 | 29.2 |
| Poultry fat | 2.89 | 2.89 | 3.21 | 3.21 | 3.84 | 3.84 |
| Limestone | 0.76 | 0.76 | 0.77 | 0.77 | 0.77 | 0.77 |
| Dicalcium phosphate | 1.28 | 1.28 | 1.23 | 1.23 | 1.21 | 1.21 |
| Salt | 0.38 | 0.38 | 0.39 | 0.39 | 0.39 | 0.39 |
| L-Lysine HCl | 0.22 | 0.22 | 0.21 | 0.21 | 0.20 | 0.20 |
| DL-Methionine | 0.33 | 0.33 | 0.27 | 0.27 | 0.23 | 0.23 |
| L-Threonine | 0.12 | 0.12 | 0.10 | 0.10 | 0.08 | 0.08 |
| L-Valine | 0.16 | 0.16 | 0.13 | 0.13 | 0.11 | 0.11 |
| Minerals^1^ | 0.10 | 0.10 | 0.10 | 0.10 | 0.10 | 0.10 |
| Vitamins^2^ | 0.08 | 0.08 | 0.08 | 0.08 | 0.08 | 0.08 |
| Coccidiostat | 0.05 | 0.05 | 0.05 | 0.05 | 0.05 | 0.05 |
| Spirulina platensis | - | 0.5 | - | 0.5 | - | 0.5 |
|  |  |  |  |  |  |  |
| Calculated Composition (% unless specified) |  |  |  |  |  |  |
| Moisture | 10.8 | 10.8 | 10.8 | 10.8 | 10.7 | 10.7 |
| Crude protein | 23.47 | 23.47 | 21.4 | 21.4 | 19.86 | 19.86 |
| ME, kcal/kg | 3000 | 3000 | 3075 | 3075 | 3150 | 3150 |
| Total calcium | 0.75 | 0.75 | 0.72 | 0.72 | 0.70 | 0.70 |
| Total phosphorus (P) | 0.64 | 0.64 | 0.61 | 0.61 | 0.59 | 0.59 |
| Available P | 0.38 | 0.38 | 0.36 | 0.36 | 0.35 | 0.35 |
| Digestible lysine | 1.28 | 1.28 | 1.15 | 1.15 | 1.05 | 1.05 |
| Digestible threonine | 0.87 | 0.87 | 0.78 | 0.78 | 0.71 | 0.71 |
| Digestible methionine | 0.66 | 0.66 | 0.58 | 0.58 | 0.52 | 0.52 |
| Digestible TSAA | 0.96 | 0.96 | 0.86 | 0.86 | 0.79 | 0.79 |
| Sodium | 0.17 | 0.17 | 0.17 | 0.17 | 0.17 | 0.17 |
| Chloride | 0.33 | 0.33 | 0.33 | 0.33 | 0.33 | 0.33 |
| DEB (mEq/kg) | 249.63 | 249.63 | 222.93 | 222.93 | 202.45 | 202.45 |
| Choline (mg/kg) | 1204 | 1204 | 1112 | 1112 | 1039 | 1039 |
|  |  |  |  |  |  |  |
| Analyzed composition |  |  |  |  |  |  |
| Crude Protein | 22.5 | 19.8 | 18.7 | 19.0 | 18.9 | 20.2 |
| ME | 4101 | 4055 | 4093 | 4067 | 4096 | 4190 |
| Fat | 6.04 | 5.53 | 5.78 | 6.42 | 5.54 | 7.36 |

^1^The mineral premix contributed (per kg of diet): manganese, 100 mg; zinc, 100 mg; calcium, 69 mg; copper, 15 mg; iron, 15 mg; iodide, 1.2 mg; selenium, 0.25 mg. ^2^The vitamin premix contributed (per kg of diet): vitamin A, 7,716 IU; vitamin D3, 5,512 ICU; vitamin E, 55 IU (49.6 mg); niacin, 38.58 mg; d-pantothenic acid, 9.92 mg; riboflavin, 6.61 mg; pyridoxine, 2.76 mg; thiamine, 1.54 mg; menadione, 1.5 mg; folic acid, 0.88 mg; biotin, 0.08 mg; vitamin B12, 0.01 mg.

**Supplementary Table 2.** Flavonoid and phenolic compounds determined by LC-MS/MS in *Spirulina platensis*.

| **Compounds** | **Molecular Formula** | **Aqueous extract (ppm)** | **Methanol extract (ppm)** | **RT**  **(min)** |
| --- | --- | --- | --- | --- |
| **Flavanoids**  Quercetin  Taxifolin  Pinostrobin  Luteolin  Sakuranetin  3,3-dimethyl quercetin  3-O-methyl quercetin  Hesperetin  Isorhamnetin  Quercetin-3-ß-glycoside  **Phenolics**  Benzoic acid  2-phenylacetic acid (Phenylacetic acid)  4-Hydroxybenzoic Acid (p-hydroxybenzoic acid (PHBA))  3,5-Dimethoxy-4-hydroxycinnamic acid (Sinapic acid)  3-Hydroxy-4-methoxybenzoic acid (isovanillic acid)  4-hydroxy-3-methoxybenzoic acid (Vanillic)  3,4,5-trihydroxybenzoic acid (gallic acid)  Hippuric Acid (2-benzamidoacetic acid)  3,4-Dihydroxybenzoic Acid (Protocatechuic acid)  2-hydroxybenzoic acid (Salicylic acid)  2-hydroxybenzoylaminoacetic acid (o-Hydroxyhippuric)  2,5 Dihydroxyphenylacetic Acid (Homogentisic acid)  3-hydroxyphenylacetic acid (m-hydroxyphenylacetic acid)  Neochlorogenic acid (-)  Chlorogenic acid | C_15_H_10_O_7_  C_15_H_12_O_7_  C_16_H_14_O_4_  C_15_H_10_O_6_  C_16_H_14_O_5_  C_17_H_14_O_7_  C_16_H_12_O_7_  C_16_H_14_O_6_  C_16_H_12_O_7_  C_21_H_20_O_12_  C_7_H_6_O_2_  C_8_H_8_O_2_  C_7_H_6_O_3_  C_11_H_12_O_5_  C_8_H_8_O_4_  C_8_H_8_O_4_  C_7_H_6_O_5_  C_9_H_9_NO_3_  C_7_H_6_O_4_  C_7_H_6_O_3_  C_9_H_9_NO_4_  C_8_H_8_O_4_  C_8_H_8_O_3_  C_16_H_18_O_9_  C_16_H_18_O_9_ | 0.0043  -  -  0.0033  -  0.0001  0.0019  -  0.0006  0.0005  24.724  35.050  0.538  -  -  -  -  0.103  -  0.115  0.022  0.077  3.184  -  0.0051 | 0.0234  0.0173  0.0058  0.0043  0.0018  0.0012  0.0011  0.0004  -  -  925.060  350.500  30.990  3.970  3.955  3.429  2.230  1.760  1.240  0.524  0.300  -  -  0.0012  - | 6.297  3.59  11.683  6.731  9.568  8.467  7.064  7.095  7.966  4.032  7.935  7.543  4.136  6.258  4.59  4.539  2.448  4.21  3.126  8.442  5.469  2.647  3.134  2.558  2.662 |

Note: RT: Retention time
